# NUDT15 codon 139 is the best pharmacogenetic marker for predicting thiopurine-induced severe adverse events in Japanese patients with inflammatory bowel disease: a multicenter study

**DOI:** 10.1007/s00535-018-1486-7

**Published:** 2018-06-19

**Authors:** Yoichi Kakuta, Yosuke Kawai, Daisuke Okamoto, Tetsuya Takagawa, Kentaro Ikeya, Hirotake Sakuraba, Atsushi Nishida, Shoko Nakagawa, Miki Miura, Takahiko Toyonaga, Kei Onodera, Masaru Shinozaki, Yoh Ishiguro, Shinta Mizuno, Masahiro Takahara, Shunichi Yanai, Ryota Hokari, Tomoo Nakagawa, Hiroshi Araki, Satoshi Motoya, Takeo Naito, Rintaro Moroi, Hisashi Shiga, Katsuya Endo, Taku Kobayashi, Makoto Naganuma, Sakiko Hiraoka, Takayuki Matsumoto, Shiro Nakamura, Hiroshi Nakase, Tadakazu Hisamatsu, Makoto Sasaki, Hiroyuki Hanai, Akira Andoh, Masao Nagasaki, Yoshitaka Kinouchi, Tooru Shimosegawa, Atsushi Masamune, Yasuo Suzuki, for the MENDEL study group

**Affiliations:** 1 Division of Gastroenterology, Tohoku University Graduate School of Medicine, 1-1 Seiryo, Aoba, Sendai, 980-8574 Japan; 2 Department of Integrative Genomics, Tohoku Medical Megabank Organization, Tohoku University, Sendai, Japan; 3 Department of Human Genetics, Graduate School of Medicine, University of Tokyo, Tokyo, Japan; 4 Division of Internal Medicine, Department of Inflammatory Bowel Disease, Hyogo College of Medicine, Nishinomiya, Japan; 5 Centre for Gastroenterology and Inflammatory Bowel Disease Research, Hamamatsu South Hospital, Hamamatsu, Japan; 6 Department of Gastroenterology and Hematology, Hirosaki University Graduate School of Medicine, Hirosaki, Japan; 7 Department of Gastroenterology, Shiga University of Medical Science, Otsu, Japan; 8 Division of Gastroenterology, Department of Internal Medicine, Aichi Medical University School of Medicine, Nagakute, Japan; 9 The Third Department of Internal Medicine, Kyorin University School of Medicine, Mitaka, Japan; 10 Center for Advanced IBD Research and Treatment, Kitasato University Kitasato Institute Hospital, Tokyo, Japan; 11 Department of Gastroenterology and Hepatology, Sapporo Medical University School of Medicine, Sapporo, Japan; 12 Department of Surgery, IMSUT Hospital, The Institute of Medical Science, The University of Tokyo, Tokyo, Japan; 13 Department of Gastroenterology and Hematology, Hirosaki National Hospital, Hirosaki, Japan; 14 Division of Gastroenterology and Hepatology, Department of Internal Medicine, Keio University School of Medicine, Tokyo, Japan; 15 Department of Gastroenterology and Hepatology, Okayama University Graduate School of Medicine, Dentistry, and Pharmaceutical Sciences, Okayama, Japan; 16 Division of Gastroenterology, Department of Internal Medicine, School of Medicine, Iwate Medical University, Morioka, Japan; 17 Division of Gastroenterology and Hepatology, Department of Internal Medicine, National Defense Medical College, Tokorozawa, Japan; 18 Department of Gastroenterology, Graduate School of Medicine, Chiba University, Chiba, Japan; 19 Division of Endscopy, Gifu University Hospital, Gifu, Japan; 20 IBD Center, Sapporo-Kosei General Hospital, Sapporo, Japan; 21 Health Administration Center, Center for the Advancement of Higher Education, Tohoku University, Sendai, Japan; 22 Department of Internal Medicine, Toho University Sakura Medical Center, Sakura, Japan

**Keywords:** NUDT15, GWAS, Thiopurine, Inflammatory bowel disease, Pharmacogenetics

## Abstract

**Background:**

Despite NUDT15 variants showing significant association with thiopurine-induced adverse events (AEs) in Asians, it remains unclear which variants of NUDT15 or whether additional genetic variants should be tested to predict AEs. To clarify the best pharmacogenetic test to be used clinically, we performed association studies of NUDT15 variants and haplotypes with AEs, genome-wide association study (GWAS) to discover additional variants, and ROC analysis to select the model to predict severe AEs.

**Methods:**

Overall, 2630 patients with inflammatory bowel disease (IBD) were enrolled and genotyped for NUDT15 codon 139; 1291 patients were treated with thiopurines. diplotypes were analyzed in 970 patients, and GWASs of AEs were performed with 1221 patients using population-optimized genotyping array and imputation.

**Results:**

We confirmed the association of NUDT15 p.Arg139Cys with leukopenia and alopecia (*p* = 2.20E−63, 1.32E−69, OR = 6.59, 12.1, respectively), and found a novel association with digestive symptoms (*p* = 6.39E−04, OR = 1.89). Time to leukopenia was significantly shorter, and when leukopenia was diagnosed, thiopurine doses were significantly lower in Arg/Cys and Cys/Cys than in Arg/Arg. In GWASs, no additional variants were found to be associated with thiopurine-induced AEs. Despite strong correlation of leukopenia frequency with estimated enzyme activities based on the diplotypes (*r*^2^ = 0.926, *p* = 0.0087), there were no significant differences in the AUCs of diplotypes from those of codon 139 to predict severe AEs (AUC = 0.916, 0.921, for acute severe leukopenia, AUC = 0.990, 0.991, for severe alopecia, respectively).

**Conclusions:**

Genotyping of NUDT15 codon 139 was sufficient to predict acute severe leukopenia and alopecia in Japanese patients with IBD.

**Electronic supplementary material:**

The online version of this article (10.1007/s00535-018-1486-7) contains supplementary material, which is available to authorized users.

## Introduction

The thiopurine drug 6-mercaptopurine (6-MP) and its pro-drug azathiopurine (AZA) are most commonly used for immunomodulatory treatments for inflammatory bowel disease (IBD), represented by ulcerative colitis (UC), Crohn’s disease (CD), and intestinal Behçet’s disease (BD) [1]. Several lines of evidence and a range of experience have shown that thiopurines are key drugs to maintain IBD remission [2, 3]. Furthermore, combination therapy using anti-TNF biologics with thiopurines reduces the formation of anti-drug antibodies and is expected to prevent the loss of response to biologics [4, 5]. Although substantial evidence of thiopurine’s efficacy has been presented, it has been reported that several types of thiopurine-induced adverse events (AEs) were experienced in association with its use, especially during the induction period. The most common but serious AE is leukopenia, so doctors need to frequently monitor white blood cell (WBC) counts during the initiation period. Although it is not life-threatening, severe alopecia may also occur, and it results in a very serious event causing cosmetic problems requiring long recovery period. Leukopenia is considered to be one of the dose-dependent AEs. However, although the standard dose of thiopurines in Japan (AZA: 1–2 mg/kg/day) is almost half that in Europe (AZA: 2–2.5 mg/kg/day), the incidences of these two serious AEs in East Asian populations are higher than that in Caucasians [6, 7].

Genetic polymorphism of thiopurine S-methyltransferase (TPMT) causing TMPT deficiency is a well-established genetic marker of thiopurine-induced leukopenia in Caucasians [8]. However, TPMT genotypes were shown not to be associated with leukopenia in the Japanese [7, 9, 10] and TPMT deficiency fails to explain the higher incidence of adverse reactions in patients with IBD in East Asia. Therefore, it was considered that there are population-specific genetic variants associated with thiopurine intolerance. The first epoch-making discovery was reported by Yang et al., who specifically conducted a genome-wide association study (GWAS) in Korean CD to reveal that a non-synonymous SNP, p.Arg139Cys (R139C), in nucleoside diphosphate-linked moiety X-type motif 15 (NUDT15) is very strongly associated with thiopurine-induced severe leukopenia [11]. Subsequently, we reported a stronger association of this SNP with severe alopecia [12]. After these reports, several replication or functional studies were performed. Moriyama et al. defined six major haplotype (*1–*6) combinations of four coding variants in exons 1 and 3; the combination of these haplotypes (diplotype) was significantly associated with tolerated 6-MP dose in children with acute lymphoblastic leukemia (ALL) among Hispanics and Asians [13]. Diplotypes were also reportedly associated with the frequencies of leukopenia in adult patients with IBD in the Chinese [14].

Despite the identification and clinical application of genetic variants of NUDT15 as pharmacogenetic markers of severe leukopenia and alopecia in IBD, the optimal approach in the clinical context with respect to genotyping of p.Arg139Cys or diplotyping of NUDT15 remains unclear.

In the present study, to determine the optimal pharmacogenetic test for predicting thiopurine-induced AEs in a clinical context, we performed not only an association study of the NUDT15 variants and their haplotypes/diplotypes, but also a GWAS to discover additional variants associated with AEs and ROC analysis to select a model to predict severe AEs.

## Materials and methods

### Study design and participants

The multicenter study for evaluation of NUDT15 genotyping efficiency to detect thiopurine-induced alopecia and leukopenia (MENDEL) was a multicenter, retrospective pharmacogenetic study. Staff at a total of 32 institutions conducted the study from December 2015 through September 2017. The eligible patients were diagnosed with CD, UC, or BD and had been treated with at least one of the following drugs: salazosulfapyridine (SASP), mesalamine (5-ASA), infliximab (IFX), adalimumab (ADA), 6-MP, and AZA. Patient diagnosis, sex, age at the time of enrollment, history of usage of these drugs, and adverse events were collected from the medical records. The definition of adverse events in this study was all clinical adverse events that triggered a modification in the usage of a drug or its discontinuation when the doctor considered the events to be associated with the drug.

**Fig. 1:**
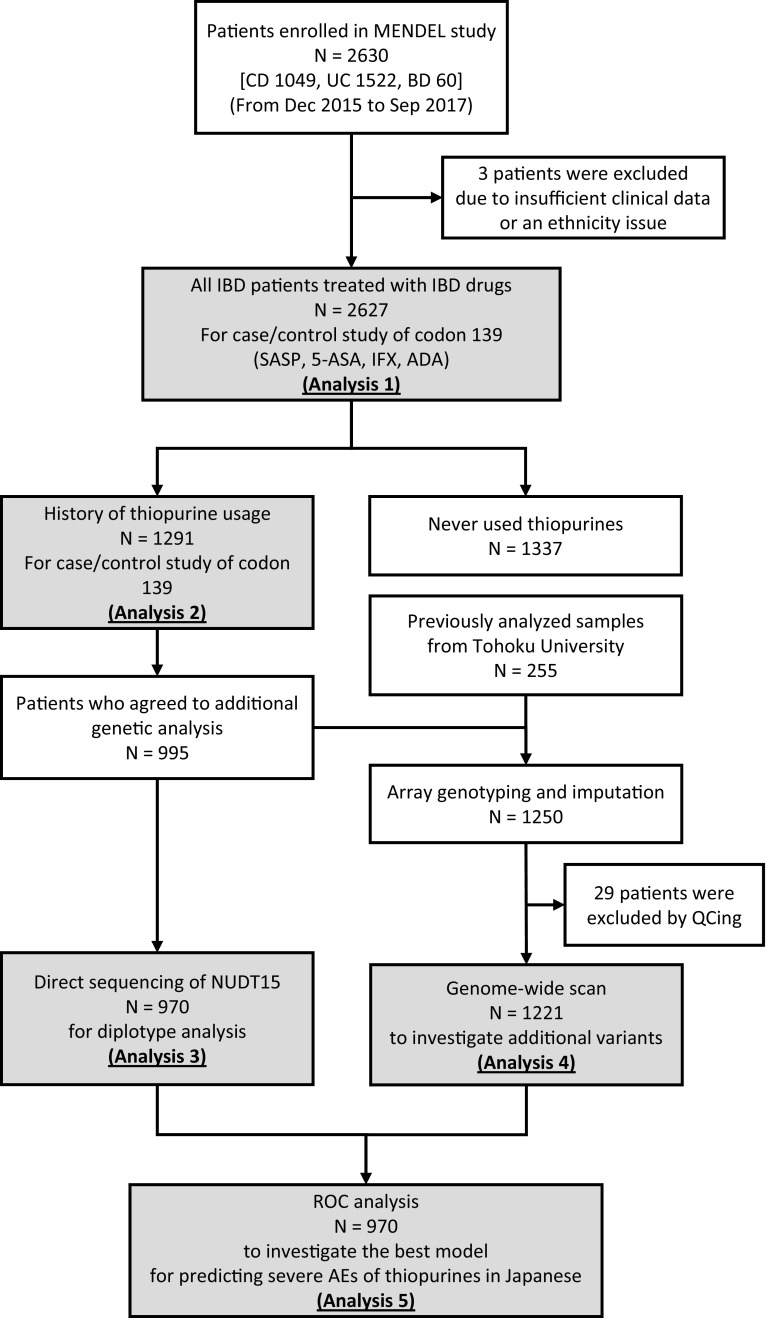
Flow of the analysis in this study. In total, 2630 patients with IBD were enrolled. All the patients were genotyped for NUDT15 codon 139. The number of patients exposed to thiopurines was 1291; additional genetic analyses were performed on 995 of these 1291 patients. This study consisted of five analyses: (analysis 1) association study of NUDT15 p.Arg139Cys with adverse events of other IBD drugs; (analysis 2) association analysis of NUDT15 codon 139 with thiopurine-induced adverse events; (analysis 3) diplotype-based association study of NUDT15 with thiopurines; (analysis 4) GWAS of thiopurine-induced adverse events; (analysis 5) ROC analysis to investigate the best model to predict thiopurine-induced severe AEs in Japanese patients with IBD

In total, 2630 IBD patients (CD 1049, UC 1522, and BD 60) were enrolled and genotyped for NUDT15 p.Arg139Cys. The study consisted of the following five association analyses: (analysis 1) association study of NUDT15 p.Arg139Cys with adverse events of other IBD drugs (analysis 2) replication and additional association analysis of NUDT15 codon 139 with all thiopurine-induced adverse events, (analysis 3) diplotype-based association study of NUDT15 with thiopurines, (analysis 4) genome-wide association study (GWAS) and replication analysis of previously reported SNPs with thiopurine-induced adverse events, and (analysis 5) ROC analysis to investigate the best model to predict thiopurine-induced severe AEs in Japanese patients with IBD (Fig. 1).

In the GWASs, we combined 255 additional samples from Tohoku University Hospital, most of which had previously been analyzed in the first replication study of NUDT15 in Japanese IBD. The protocol of the study was approved by the institutional review board at each institution; all adult patients provided written informed consent, and if the patients were minors (< 20 years old), parental consent was obtained.

### Genotyping of NUDT15 codon 139

Genomic DNA was extracted from peripheral blood leukocytes by standard phenol–chloroform precipitation. Three common genotypes of NUDT15 codon 139 were determined by the genotyping of rs116855232 (c.415C > T, Arg139Cys) using a TaqMan SNP genotyping assay, as previously described [12], at the central laboratory, LSI Medience Corporation (Tokyo, Japan). The outliers of three clusters (c.415 CC, CT, or TT) were analyzed by direct sequencing, by the same method as described below.

### Direct sequencing of NUDT15 coding regions

Exons 1, 2, and 3 of NUDT15 were analyzed by capillary electrophoresis Sanger sequencing methods. Amplification primers are summarized in Supplementary Table 1. Each 10 μL PCR amplification reaction contained 4 ng of gDNA, 1.2 pmol each primer, and 5 μL of BigDye^®^ Direct PCR Master Mix. The reactions were amplified using a DNA Engine PTC-200 (MJ Research, NV, USA). The PCR conditions were as follows: initial holding at 95 °C for 10 min, 35 cycles of denaturing at 9 °C for 3 s, annealing at 62 °C for 10 s, and extension at 68 °C for 30 s, and post-extension at 72 °C for 2 min. Each 10 μL sequencing reaction contained 7 μL of PCR product, 2 μL of BigDye^®^ Direct Sequencing Master Mix, and 1 μL of BigDye^®^ Direct M13 Fwd or M13 Rev sequencing primer. After initial incubation at 37 °C for 15 min and 80 °C for 2 min, cycling conditions were as follows: 96 °C for 1 min, and then 25 cycles of sequencing at 96 °C for 10 s, 50 °C for 5 s, and 60 °C for 75 s. At completion of the sequencing reaction, the sequencing products were purified using the BigDye XTerminator^®^ Purification Kit. Electrophoresis was performed on an Applied Biosystems^®^ 3500xL Genetic Analyzer with POP-7™ Polymer.

### Genome-wide SNP genotyping and imputation

Genome-wide SNP genotypes were determined using Japonica array [15]. For quality control (QC), SNPs with call rates < 97% or with a malformed genotype cluster according to SNPolisher program (Thermofisher) as well as samples with genotyping rates < 97%, samples of cryptic relatives (PI_HAT > 0.5), or samples that were identified as outliers upon plotting of the first two components of principal component analysis were excluded from the GWASs. Untyped genotypes were imputed in the GWAS samples with the haplotype reference panel of 2,036 individuals from Japan (2KJPN panel, which includes > 20 million SNVs [16]) [1, 15, 16]. QC-passed SNPs were prephased using EAGLE (v 2.4) with the default settings [17]. Phased genotypes were imputed with IMPUTE4 (v 1.0) using the 2KJPN panel as follows: Ne 20000 buffer 500, and data for 24,344,327 variants were obtained [18]. SNVs with low imputation quality (with a posterior genotype probability of < 0.8 for each genotype or with info score < 0.5 for each variant), low call rate of < 0.97, minor allele frequency of < 0.01 or 0.05 (depends on the number of cases, 0.01 for GWASs of > 50 cases, 0.05 for GWASs of low-frequency AEs or conditional GWASs on rs116855232), and Hardy–Weinberg equilibrium (HWE) *p* < 1 × 10^−6^ were excluded. Further analysis was performed using genotype data from > 5 million SNVs from 1,221 Japanese IBD cases.

### Haplotype and diplotype phasing

Diplotypes, namely, combinations of haplotypes in each individual, were inferred from the genotype information of the NUDT15 locus by applying a statistical phasing approach. For the samples from the MENDEL study, the genotypes at the NUDT15 locus obtained by Japonica array combined with the results obtained by exon 1–3 sequencing were phased by EAGLE (v 2.4). For the 2,036 samples from the general population (ToMMo cohort) [16, 19], the variants of the NUDT15 locus were discovered from the mapped reads of whole-genome sequencing with the HaplotypeCaller program in GATK and the resultant genotypes were phased with EAGLE. We followed the six major haplotype definitions (*1–*6) by Moriyama et al., and we defined haplotypes with three additional variants reported recently, p.Arg34Thr, p.Lys35Glu, and p.Gly17_Val18del, as *7, *8, and *9 following Moriyama’s nomenclatures (Supplementary Fig. S1).

### Statistical analysis

Categorical variables of clinical phenotypes were compared using Fisher’s exact test. Associations of genotype frequencies with adverse events were analyzed by Cochran–Armitage trend analysis, while those of allele frequencies were analyzed by Chi-squared test. GWASs were evaluated using logistic regression with gender as a covariate using the PLINK v 1.90 software [20]. SNPs with a *p* value < 1 × 10^−8^ were considered to have genome-wide significance, while those with a *p* value < 1 × 10^−6^ were considered to be nominally significant. SNPs located within 250 kbp of one another were considered to be in one region. Regional association plots were generated using the LocusZoom application [21]. We generated a conditional logistic model with pairs of each SNP and rs116855232 (NUDT15 p.Arg139Cys) to determine whether one or more causal SNPs might explain the observed association. *p* values are interpretable as the residual variation explained by the SNP, conditional on the inclusion of rs116855232. Receiver operating characteristic (ROC) curve analysis was performed using the R package pROC. All statistical analyses, except genome-wide logistic regression, were performed using the R software (v 3.4.4) (http://www.r-project.org/).

## Results

### Association analyses of the genotypes of NUDT15 codon139 with thiopurine-induced AEs

#### Thiopurine-induced digestive symptoms were significantly associated with Arg139Cys

Of 2630 enrolled patients, three patients were excluded due to insufficient clinical data or an ethnicity issue (non-Japanese). Finally, a total of 2627 patients were included in analysis 1; the patient characteristics and genotype frequencies of NUDT15 codon 139 are summarized in Supplementary Table 2. We confirmed the strong and significant associations of NUDT15 p.Arg139Cys with AEs derived from thiopurines (*p* = 1.55E−36, OR = 4.13). However, AEs from four other IBD drugs were not significantly associated (Supplementary Table 3). A total of 1291 patients had a history of thiopurine usage, and 460 patients had discontinued or modified their usage of thiopurine due to AEs. The breakdown of AEs is summarized in Supplementary Table 4. No significant differences in the frequencies of each type of AE were observed between the diseases. As previously reported, we confirmed the strong associations of NUDT15 p.Arg139Cys with leukopenia and alopecia (*p* = 2.20E−63 and 1.32E−69, OR = 6.59 and 12.1, respectively), and we newly identified its significant association with thiopurine-induced digestive symptoms (*p* = 6.39E−04, OR = 1.89) (Table 1).

**Table 1:** Associations between genotypes of codon 139 and adverse events associated with thiopurines

Codon 139 genotype^a^	Arg/Arg	Arg/Cys	Cys/Cys	*p* values^b^	Allelic association^c^ (Arg vs. Cys)	
					*p* values	OR (95% CI)
Number of subjects	958	275	49			
All adverse events of thiopurines	260 (27.1%)	141 (51.3%)	49 (100.0%)	1.29E−32	1.55E−36	4.13 (3.28–5.20)
Leukopenia	94 (9.8%)	94 (34.2%)	45 (91.8%)	2.00E−56	2.20E−63	6.59 (5.19–8.36)
Severe (WBC < 2000/μL)	17 (1.8%)	33 (12.0%)	38 (77.6%)	3.09E−67	2.56E−75	13.1 (9.41–18.2)
Acute (< 8 weeks)	14 (1.5%)	27 (9.8%)	39 (79.6%)	7.06E−73	1.70E−80	15.6 (11.0–22.1)
Acute and severe	3 (0.3%)	11 (4.0%)	33 (67.3%)	2.62E−72	4.23E−80	34.2 (20.0–58.7)
Alopecia	28 (2.9%)	13 (4.7%)	46 (93.9%)	3.48E−62	1.32E−69	12.1 (8.67–16.8)
Severe (objective)	1 (0.1%)	3 (1.1%)	44 (89.8%)	8.51E−101	6.61E−113	141 (56.9–350)
Mild (self-reported)	27 (2.8%)	10 (3.6%)	2 (4.1%)	4.17E−01	4.83E−01	
Liver dysfunction	38 (4.0%)	8 (2.9%)	1 (2.0%)	3.04E−01	3.44E−01	
Pancreatitis	18 (1.9%)	2 (0.7%)	0 (0.0%)	1.06E−01	1.34E−01	
Digestive symptoms	55 (5.7%)	31 (11.3%)	6 (12.2%)	9.55E−04	6.39E−04	1.89 (1.32–2.72)
Infection	11 (1.1%)	4 (1.5%)	2 (4.1%)	1.61E−01	2.11E−01	
Fever	10 (1.0%)	3 (1.1%)	0 (0.0%)	6.82E−01	8.75E−01	
Skin symptom	5 (0.5%)	2 (0.7%)	0 (0.0%)	9.79E−01	1.00	
Malignant tumor	1 (0.1%)	0 (0.0%)	1 (2.0%)	5.91E−02	1.93E−01	

^a^Rare genotypes (CH and RH) were excluded

^b^Cochran–Armitage trend analysis

^c^Chi-squared test

#### Time to leukopenia is significantly shorter in Arg/Cys and Cys/Cys than in Arg/Arg

**Fig. 2:**
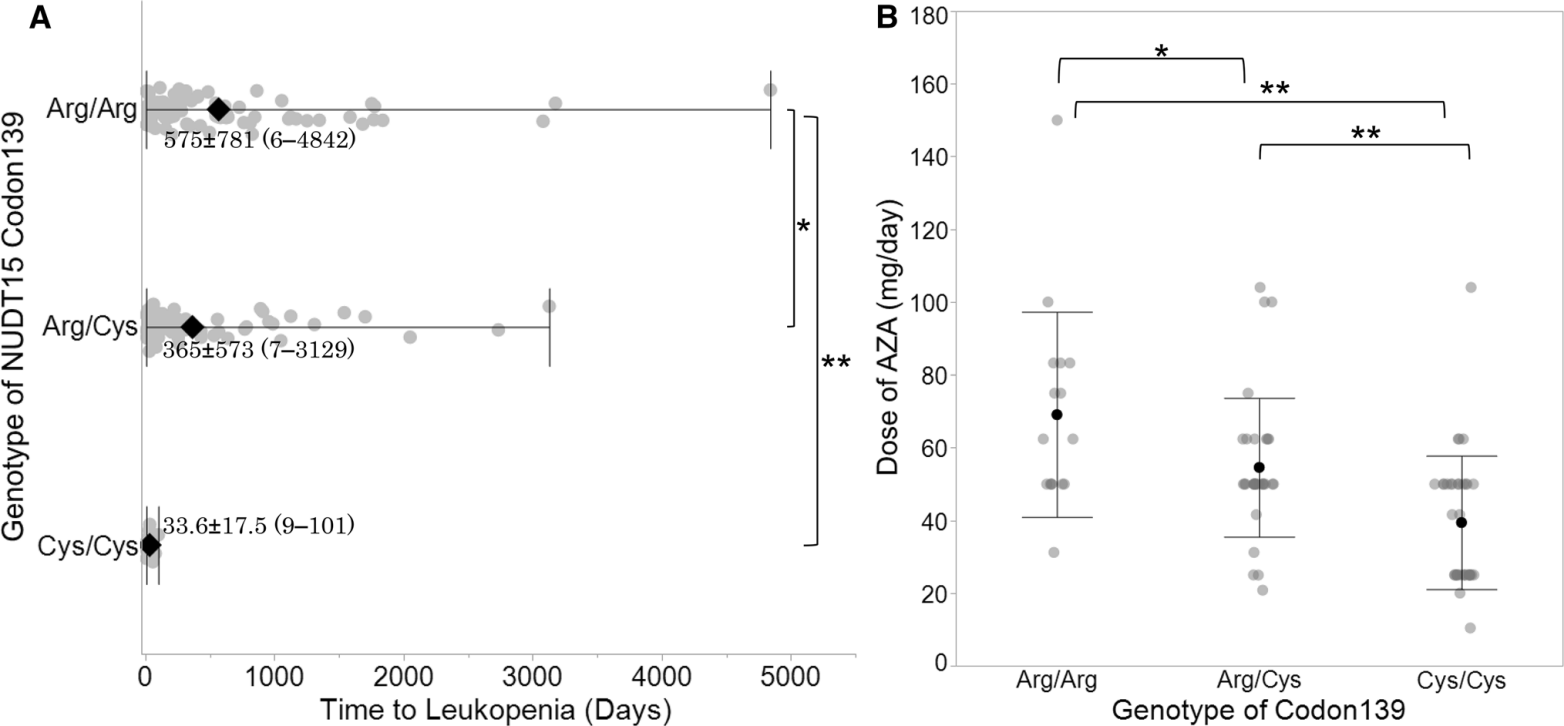
Time to leukopenia and the doses of AZA according to the genotype of NUDT15 codon 139. **a** Time to leukopenia after starting treatment with thiopurines is plotted in terms of the genotype. Average time to leukopenia in patients with the Cys/Cys and Arg/Cys genotypes was significantly shorter than that for those with the Arg/Arg genotype. **b** Doses of thiopurines at the time when severe leukopenia was diagnosed were plotted. The 6-MP dose was adjusted to AZA equivalents by multiplying by 2.08. The dose of the patients carrying the p.Arg139Cys allele was significantly lower than that of the patients with Arg/Arg. *p < 0.05, **p < 0.005

The data of the time to leukopenia (WBC < 3000/μL) were available in 211 of 236 patients who experienced leukopenia. The average time to leukopenia in the patients with the Cys/Cys or Arg/Cys genotype was significantly shorter than that in Arg/Arg (33.6 ± 17.5, 365 ± 573, 575 ± 781 days, *p* = 3.51E−02, 1.45E−05, respectively) (Fig. 2a). The doses of thiopurines at the time when severe leukopenia was diagnosed were 54.6 ± 19.1 mg/day in Arg/Cys and 39.4 ± 3.1 mg/day in Cys/Cys, which were significantly lower than 69.1 ± 28.1 mg/day in Arg/Arg (*p* = 2.46E−02 and 8.50E−06, respectively) (Fig. 2b).

#### Rare genotype of Cys/His is a risk for acute severe leukopenia, but not for alopecia

The results of logistic regression analysis of severe AEs with all genotypes of codon 139 including rare histidine allele are summarized in Table 2. In comparison with the wild-type genotype (Arg/Arg), the odds ratios for acute severe leukopenia were higher in the order of Arg/Cys, Cys/His, and Cys/Cys (OR = 13.4, 318, and 807, respectively), while those for severe alopecia were higher in the order of Arg/Cys and Cys/Cys (OR = 106 and 8421, respectively). Arg/His was not a risk for AEs, while Cys/His was a risk for acute severe leukopenia (WBC < 2000/μL, < 8 weeks) but not for severe alopecia.

**Table 2:** Associations between genotype of codon 139 and severe adverse events associated with thiopurines

Genotype	*N*	All AEs	Acute severe leukopenia (WBC < 2000/μL, < 8 weeks)			Severe alopecia		
		Frequencies (%)	Frequencies (%)	*p* value*	OR (95% CI)	Frequencies (%)	*p* value*	OR (95% CI)
Arg/Arg (RR)	958	258 (26.9%)	3 (0.3%)	(reference)	1.0	1 (0.1%)	(reference)	1.0
Arg/His (RH)	7	2 (28.6%)	0 (0%)	9.90E−01	–	0 (0%)	9.94E−01	–
Arg/Cys (RC)	275	135 (49.1%)	11 (4.0%)	7.43E−05	13.4 (3.7–48.4)	3 (1.1%)	4.16E−02	10.6 (1.1–101)
Cys/His (CH)	2	2 (100.0%)	1 (50.0%)	1.62E−04	318 (15.9–6352)	0 (0%)	9.97E−01	–
Cys/Cys (CC)	49	49 (100.0%)	33 (67.3%)	7.30E−24	807 (219–2969)	44 (89.8%)	3.07E−16	8421 (963–73625)

*CI* confidence interval

*Logistic regression test

### Association analyses of NUDT15 haplotypes/diplotypes with leukopenia and alopecia

#### Rare non-synonymous variants were newly identified in the Japanese population

By direct sequencing of exons 1, 2, and 3 of the NUDT15 gene, we identified in Japanese the rare haplotype *9 containing the p.Gly17_Val18del variant, which had previously been reported only in a non-Asian population [22]. No other additional novel functional variants were identified. In addition, we analyzed NUDT15 regions of 2KJPN using the whole-genome sequencing results of a residential cohort of 2036 Japanese. Haplotype *9 was also found in 2KJPN, and we identified two novel haplotypes containing p.Met1Thr (loss of start codon) and p.Gly47Arg. All of these haplotype structures and frequencies are summarized in Supplementary Fig. S1. Rare haplotypes *7 and *8 reported previously were not found in our samples or the 2KJPN data set [22].

#### Estimated enzyme activities based on the diplotypes were significantly associated with the frequencies of leukopenia

Leukopenia and alopecia frequencies in each phased diplotype are summarized in Table 3. To evaluate the associations of diplotypes with AEs, we categorized them into the five groups according to the estimated enzyme activities. Frequencies of leukopenia (WBC < 3000/μL) were 10.2% in the normal and normal (NN) group, 17.9% in the NI (normal and intermediate) group, 30.7% in the normal and low (NL) group, 60.0% in the IL group, and 94.3% in the LL group. There was a strong correlation of the frequencies of leukopenia (WBC < 3000/μL) with estimated enzyme activities (*r*^2^ = 0.926, *p* = 0.0087), but not of acute severe leukopenia or severe alopecia.

**Table 3:** Frequencies of adverse events by diplotype of NUDT15 and linear correlation to enzyme activity

Diplotype	Estimated enzyme activity	Frequencies (2KJPN)	Frequencies (MENDEL)	Leukopenia (WBC < 3000/μL)	Severe leukopenia (WBC < 2000/μL)	Acute severe leukopenia (WBC < 2000/μL, < 8 weeks)	Severe alopecia
*1*1	NN	1615 (79.3%)	697 (71.9%)	71 (10.2%)	12 (1.7%)	3 (0.4%)	0 (0.0%)
*1*2	NL	111 (5.5%)	79 (8.1%)	31 (39.2%)	8 (10.1%)	1 (1.3%)	1 (1.3%)
*1*3	NL	248 (12.2%)	125 (12.9%)	31 (24.8%)	16 (12.8%)	9 (7.2%)	2 (1.6%)
*1*4	NI	1 (0.049%)	7 (0.72%)	1 (14.3%)	0 (0.0%)	0 (0.0%)	0 (0.0%)
*1*5	NI	30 (1.5%)	17 (1.8%)	4 (23.5%)	1 (5.9%)	0 (0.0%)	0 (0.0%)
*1*6	NI	4 (0.20%)	4 (0.41%)	0 (0.0%)	0 (0.0%)	0 (0.0%)	0 (0.0%)
*1*9	NL	0 (0.0%)	1 (0.10%)	1 (100.0%)	0 (0.0%)	0 (0.0%)	0 (0.0%)
*1*10	–	1 (0.049%)	0 (0.0%)	–	–	–	–
*2*2	LL	3 (0.15%)	2 (0.21%)	2 (100.0%)	2 (100.0%)	2 (100.0%)	2 (100.0%)
*2*3	LL	7 (0.34%)	16 (1.6%)	16 (100.0%)	15 (93.8%)	14 (93.3%)	16 (100.0%)
*2*4	IL	0 (0.0%)	1 (0.10%)	1 (100.0%)	1 (100.0%)	1 (100.0%)	0 (0.0%)
*2*5	IL	0 (0.0%)	1 (0.10%)	0 (0.0%)	0 (0.0%)	0 (0.0%)	0 (0.0%)
*2*9	LL	1 (0.049%)	0 (0.0%)	–	–	–	–
*2*11	–	1 (0.049%)	0 (0.0%)	–	–	–	–
*3*3	LL	12 (0.59%)	17 (1.8%)	15 (88.2%)	11 (64.7%)	9 (60.0%)	16 (94.1%)
*3*4	IL	0 (0.0%)	1 (0.10%)	1 (100.0%)	1 (100.0%)	0 (0.0%)	0 (0.0%)
*3*5	IL	1 (0.049%)	2 (0.21%)	1 (50.0%)	0 (0.0%)	0 (0.0%)	0 (0.0%)
*5*5	II	1 (0.049%)	0 (0.0%)	–	–	–	–
	NN		697 (71.86%)	71 (10.2%)	12 (1.7%)	3 (0.4%)	0 (0.0%)
	NI		28 (2.89%)	5 (17.9%)	1 (3.6%)	0 (0.0%)	0 (0.0%)
	NL		205 (21.13%)	63 (30.7%)	24 (11.7%)	10 (4.9%)	3 (1.5%)
	IL		5 (0.52%)	3 (60.0%)	2 (40.0%)	1 (20.0%)	0 (0.0%)
	LL		35 (3.61%)	33 (94.3%)	28 (80.0%)	25 (71.4%)	34 (97.1%)
	Correlation analysis		*r* ^2^	**0.926**	**0.847**	0.718	0.504
			*p* value	**0.0087**	**0.027**	0.07	0.18

Bold numbers indicate the significant correlations (*p*-values < 0.05)

*NN* normal and normal (1*1), *NI* normal and intermediate (*1*4, *1*5, *1*6), *NL* normal and low (*1*2, *1*3, *1*9), *IL* intermediate and low (*2*4, *2*5, *3*4, *3*5), *LL* low and low (*2*2, *2*3, *3*3)

### Only NUDT15 was significantly associated with severe leukopenia and alopecia

**Fig. 3:**
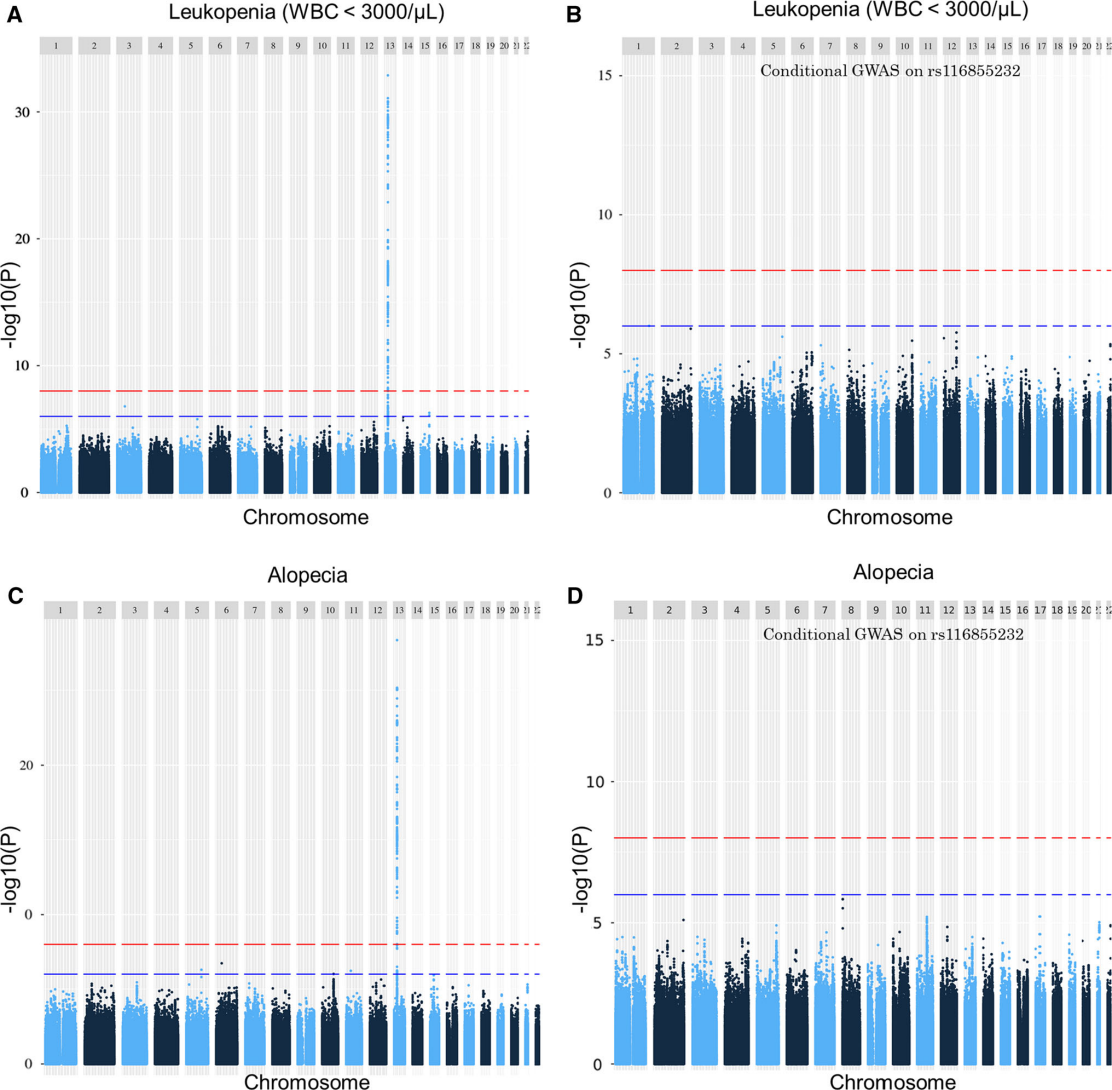
Manhattan plots for results of the discovery and conditional GWASs for thiopurine-induced leukopenia and alopecia in Japanese individuals. Single-nucleotide polymorphisms are plotted according to chromosomal location, with the − log_10_(P) from the results of GWASs. The red line indicates the threshold for genome-wide significance (*p* = 1E−8). The blue line indicates the threshold for nominal significance (*p* = 1E−6). **a** GWAS for thiopurine-induced leukopenia, **b** conditional GWAS for leukopenia on rs116855232 (p.Arg139Cys), **c** GWAS for alopecia, and **d** conditional GWAS for alopecia on rs116855232. All significant associations disappeared in the conditional GWASs

A significant association signal at chromosome 13 was observed in the results of GWAS for thiopurine-induced leukopenia and alopecia; the top hit SNP was rs116855232, NUDT15 Arg139Cys (Supplementary Table 5, Fig. 3a, c, Supplementary Figs. S2, S3a). The associations of rs116855232 with these two kinds of AEs were so strong that we performed conditional GWAS on rs116855232. Candidate regions showing nominal significance are summarized in Supplementary Table 6. All associations with leukopenia and alopecia observed in the first GWAS disappeared in the conditional analysis in leukopenia (Fig. 3b, d, Supplementary Fig. S3b). In the newly identified candidates, there were no non-synonymous functional variants.

From the GWAS results, we picked up the variants that were previously reported to be associated with thiopurine-induced leukopenia [23–26]. ABCC4 Glu757Lys showed an association with leukopenia, and rs2834826 located upstream of RUNX1 showed an association in the conditional analysis on NUDT15 Arg139Cys. TPMT Tyr240Cys (TPMT*3C) was not associated with leukopenia, as previously reported in East Asians including the Japanese (Supplementary Table 7).

We also performed GWAS for other thiopurine-induced AEs, pancreatitis, infection, digestive symptoms, liver dysfunction, skin symptoms, and fever (Supplementary Fig. S4). Candidate regions are summarized in Supplementary Table 5. rs4437130, located upstream of the CTCN4 (contactin 4) gene, and rs62561366, located upstream of PTCH1 (patched 1), were nominally associated with thiopurine-induced pancreatitis (Supplementary Fig. S5). In addition, rs12035735 in the intron of LRRC8D (leucine-rich repeat-containing 8 family, member D) was nominally associated with digestive symptoms. No significant genetic association with other AEs was observed.

### Genotyping of NUDT15 codon 139 is the best way to predict severe leukopenia and alopecia

No additional SNPs were found in the conditional GWAS; therefore, we included the genotype of NUDT15 codon 139 or NUDT15 haplotype, ABCC4, and RUNX1 in the logistic regression models to predict thiopurine-induced severe leukopenia and alopecia. To compare the genotyping of codon 139 with other models, AUCs of each model to predict the AEs were evaluated. The AUC of the NUDT15 haplotype was significantly better to predict leukopenia (WBC < 3000/μL) than the model of NUDT15 codon 139 only (AUC = 0.706 and 0.722, *p* = 0.013, respectively) (Table 4, Supplementary Fig. S6). However, the AUC of codon 139 only was better than the haplotype to predict acute severe leukopenia (AUC = 0.921 and 0.916, respectively) and severe alopecia (AUC = 0991 and 0.990, respectively). The models containing ABCC4 and/or RUNX1 showed better AUCs; however, there were no significant differences between the model of only codon 139 and other models to predict severe AEs.

**Table 4:** Comparison of logistic regression models to predict thiopurine-induced leukopenia and severe alopecia

Phenotype	Leukopenia (WBC < 3000/μL)		Severe leukopenia (WBC < 2000/μL)		Acute severe leukopenia		Severe alopecia	
Model	AUC (95%CI)	*p* value*	AUC (95 %CI)	*p* value*	AUC (95%CI)	*p* value*	AUC (95%CI)	*p* value*
NUDT15_codon139	0.706 (0.665–0.748)	NA	0.841 (0.786–0.896)	NA	0.921 (0.869–0.972)	NA	0.991 (0.981–1.000)	NA
NUDT15_haplotype	0.722 (0.680–0.764)	**0.013**	0.843 (0.789–0.898)	0.706	0.916 (0.864–0.967)	0.168	0.990 (0.979–1.000)	0.972
NUDT15_codon139 + ABCC4	0.724 (0.681–0.767)	0.555	0.839 (0.777–0.901)	0.966	0.924 (0.871–0.978)	0.925		
NUDT15_codon139 + RUNX1	0.735 (0.691–0.779)	0.358	0.858 (0.804–0.912)	0.665	0.927 (0.876–0.979)	0.858		
NUDT15_codon139 + ABCC4 + RUNX1	0.744 (0.701–0.787)	0.217	0.856 (0.798–0.913)	0.718	0.931 (0.879–0.983)	0.790		
NUDT15_haplotype + ABCC4	0.736 (0.693–0.780)	0.327	0.844 (0.783–0.904)	0.949	0.923 (0.870–0.976)	0.963		
NUDT15_haplotype + RUNX1	0.746 (0.702–0.790)	0.194	0.861 (0.807–0.914)	0.615	0.927 (0.876–0.978)	0.870		
NUDT15_haplotype + ABCC4 + RUNX1	0.753 (0.710–0.796)	0.127	0.859 (0.803–0.915)	0.645	0.931 (0.881–0.982)	0.775		

Bold number indicate the significant correlations (*p*-value < 0.05)

*p values for comparison with AUC of NUDT15_codon139

Moreover, we made eight additional models and performed ROC analyses to investigate the binary cut-off values of the codon 139 model and the haplotype models. Similar to the results of the logistic regression models, the best AUCs were diplotype_A or B models (cutoff as negative = *1*1 or *1*1*, *1*4, *1*5, *1*6, sensitivity = 0.594 and 0.566, specificity = 0.787 and 0.816, AUC = 0.691 and 0.691, respectively) to predict mild-to-severe leukopenia (WBC < 3000). The best AUCs were the Codon139_B model for severe leukopenia and acute severe leukopenia (cutoff as negative = Arg/Arg and Arg/His, sensitivity = 0.806 and 0.923, specificity = 0.790 and 0.780, and AUC = 0.798 and 0.852, respectively), and it was the Codon139_D model for severe alopecia (cutoff as positive = Cys/Cys, sensitivity = 0.919, specificity = 0.999, and AUC = 0.959) (Supplementary Table 8).

## Discussion

In this study, we first replicated the association of NUDT15 Arg139Cys with thiopurine-induced leukopenia and alopecia, and found a novel association with digestive symptoms using a large case–control dataset from more than 30 institutions in Japan. By direct sequencing analysis and reanalysis of 2KJPN data in silico, three rare non-synonymous variants of NUDT15 were newly identified in the Japanese population. We performed pharmacogenetic GWAS of thiopurine-induced AEs using a population-optimized genotyping array and imputation; the obtained results suggested that the effect of genetic background was observed only in leukopenia and alopecia. Finally, we evaluated several predictive models; the simple model based on only NUDT15 codon 139 was found to be sufficient to predict severe leukopenia and alopecia in the Japanese.

We collected DNA samples associated with cases of AEs caused by IBD drugs from all over Japan. During the study of the TaqMan assay for NUDT15 p.Arg139Cys, we identified the existence of p.Arg139His; it was rare but not negligible for the assay. Therefore, we performed an association study of NUDT15 codon 139 with AEs from several IBD drugs, and we confirmed that the association was observed only for thiopurine-induced AEs, leukocytopenia and alopecia. In addition, we newly identified the association of p.Arg139Cys with thiopurine-induced digestive symptoms. The digestive symptoms were mostly nausea or vomiting, which are considered as allergic reactions to thiopurines. However, the nausea may have heterogeneous etiologies; NUDT15 deficiency-induced leukopenia may cause nausea. It is difficult to clarify the etiology of nausea and determine that the symptoms are caused directly by p.Arg139Cys. However, we confirmed the association of digestive symptoms even after excluding all leukopenia cases [6.8% in Arg/Arg and 14.0% in Arg/Cys, *p* = 6.20E−03, OR = 2.22 (1.28–3.86)]; this may be one line of evidence, suggesting that NUDT15 variants are directly associated with digestive symptoms.

Moriyama et al. defined six major haplotype (*1–*6) combinations of four coding variants in exons 1 and 3 [13]; moreover, recently, three additional variants in exon 1 were reported [22] and we defined the additional haplotypes including these variants as *7, *8, and *9. Of these haplotypes, NUDT15 p.G17_V18del (*9) was only observed in those of European and African ancestry [22]. In this study, we found two *9 carriers: one in the MENDEL study (diplotype *1/*9) and the other in the 2KJPN cohort (diplotype *2/*9). This is the first report describing the identification of p.G17_V18del in a Japanese population. In addition, we found two additional variants, p.Met1Thr (loss of start codon) and Gly47Arg, and haplotypes carrying these variants (haplotypes defined as *10 and *11, respectively) in 2KJPN whole-genome sequencing data [16] (Supplementary Fig. S1).

The incidence of AEs in each diplotype, the combination of haplotypes in each individual, is more important information to predict the AEs in a clinical setting. A recent report described associations of leukopenia with the NUDT15 diplotype in Chinese patients with IBD [14]. In that report, the frequency of leukopenia was clearly related to NUDT15 enzyme activity based on diplotypes. We observed similar results in diplotype analysis; namely, the diplotype-based estimated enzyme activities were significantly correlated with the frequencies of leukopenia. However, diplotypes with haplotypes carrying only exon 1 variants (*5, *6, and *9) were rare, and frequencies of severe AEs, acute severe leukopenia, or severe alopecia, in these diplotypes (*1*5, *1*6, *1*9, *2*5, and *3*5), were all 0%. Therefore, diplotype analysis will be helpful to predict mild leukopenia, but there is no additional effect to predict severe AEs.

This is the first genome-wide pharmacogenetic association study of thiopurine-induced AEs in the Japanese, using a population-optimized array and imputation. JPA is specific for the Japanese population; we can easily obtain whole-genome imputation data by imputing JPA results in combination with a large number of haplotypes of more than 2000 individuals with Japanese ancestry (2KJPN). The top hit of GWASs with leukopenia and alopecia was rs116855232 (NUDT15 p.Arg139Cys). JPA does not contain the probes for rs116855232, so the genotypes were imputed. The results were compatible with our association results obtained by TaqMan assays. It demonstrated the efficacy of whole-genome imputation, population-optimized array, and imputation with population-matched reference data.

Because the associations of rs116855232 with leukopenia and alopecia were robust and very strong, we performed conditional GWASs on rs116855232 to find additional variants besides NUDT15. No other additional variants were significantly associated with these two kinds of AEs. Several genes were reported to be associated with leukopenia before and after the discovery of an association with NUDT15. TPMT, thiopurine S-methyltransferase, is well known to inactivate thiopurines by methylation and its genetic variation is one of the few pharmacogenetic predictors used in a clinical setting in the Caucasian population. TPMT*3C was reported to be significantly associated with acute leukopenia in Korean IBD [11], but no association was observed in the previous reports on studies in the Japanese. In this study, there was no association of TPMT*3C with leukopenia in our large-scale Japanese IBD data, supporting the previous results in the Japanese. ATP-binding cassette subfamily C member 4 (ABCC4), also known as multidrug-resistance protein 4 (MRP4), is associated with thiopurine metabolism; its genetic variant rs3765534 (p.Glu757Lys) was reported to be associated with leukopenia in the Japanese [23]. Runt-related transcription factor 1 (RUNX1) was reported to play an important role in hematopoiesis, and rs2834826 located upstream of this gene was found to be significantly associated with leukopenia in Korean IBD [26]. We confirmed associations of these two variants in this study, but these effect sizes were relatively small.

In the Caucasian population, thiopurine-induced pancreatitis was reported to be associated with the HLA-DQA1*02:01-HLA-DRB1*07:01 haplotype [27]. In our results, there was no signal at HLA regions at all. Two candidate regions were observed. One is located upstream of CNTN4; the variant located near this gene was reported to be a risk factor for pancreatic cancer [28]. The other is located upstream of PTCH1, coding the receptor for sonic hedgehog. The sonic hedgehog signaling pathway was reported to be associated with the development of chronic pancreatitis [29, 30]. However, how these SNPs affect the function or expression of these genes remained unknown, and all of the GWASs of AEs except leukopenia were performed with a very small sample size. Further investigation with larger sample set will be needed (Supplementary Table 9).

AEs caused by NUDT15 deficiency are very problematic in a clinical setting; severe leukopenia can be a critical event and severe alopecia affects the QOL of patients for a prolonged period. Not only could avoiding these NUDT15-derived AEs help the patients with a risk genotype of NUDT15 to not encounter severe AEs, but also for other patients, thiopurines will be more acceptable by relieving the anxiety associated with severe AEs. Therefore, testing the genotype or diplotype of NUDT15 is promising for clinical applications to predict thiopurine toxicity in Asian and Hispanic populations. Considering NUDT15 testing in a clinical context, whether to test the genotype or the diplotype is a major issue. Genotyping of codon 139 is not so difficult, and just one or two (p.Arg139His) assays are required. However, to determine the diplotype, combined analysis with two variants in exon 1 is required. In our results, the testing of NUDT15 codon 139 (p.Arg139Cys/His) showed higher AUCs than detecting the diplotype of NUDT15 to predict severe leukopenia and alopecia. However, Chao et al. reported that the predictive sensitivity of NUDT15 p.Arg139Cys for leukopenia was 49.2% in their Chinese IBD cohort, but to determine the diplotype by detecting haplotypes, *5 and *6 could increase the sensitivity to 55.4% [14]. These results suggest a discrepancy, but it may be caused by inconsistent definitions of leukopenia used. In the study, the definition of leukopenia was a WBC count of < 3500/μL, which is higher than in other reports. In our study, to predict conditions ranging from mild to severe leukopenia (WBC < 3000/μL), detection of the diplotype showed a better AUC than detection of the genotype of codon 139, which is compatible with the findings described in their report. It is important to decide how severe adverse events need to be distinguished by a clinical examination. Depending on the purpose of thiopurine usage, the target should differ among the diseases. In IBD patients, thiopurines are used in maintenance therapy. For clinical application, it is sufficient for the test to avoid severe leukopenia and alopecia. Therefore, testing of codon 139 would be the optimal clinical examination considering the time, effort, and costs associated with genotyping. If the WBC count needs to be tightly controlled, the test for detecting the diplotype should be considered.

It has been reported that NUDT15 converted thiopurine active metabolites 6-thio-GTP and 6-thio-dGTP to 6-thio-GMP and 6-thio-dGMP [13, 31]. The variants of NUDT15 were shown to exert lower enzyme activity causing a higher thiopurine active metabolite level, thereby resulting in dose-dependent AEs such as thiopurine-induced leukopenia and alopecia. In our results, the doses of thiopurines at the time when severe leukopenia was diagnosed were lower and the time to leukopenia was shorter in the patients with the genotypes of Arg/Cys and Cys/Cys. These results suggest that AEs associated with NUDT15 deficiency were dose-dependent, and the optimal dose must differ in each genotype. No patients with the Arg/His genotype experienced severe leukopenia, but both the two patients with the Cys/His genotype experienced severe or mild leukopenia at the AZA dose of 25 mg/day. Previously, we reported that the average maintenance doses of thiopurines are 1.03 mg/kg/day in patients with the Arg/Arg genotype and 0.574 mg/kg/day in those with the Arg/Cys genotype [12]. We analyzed the correlation between the 6-MP doses and time to leukopenia in the patients with the Cys/Cys genotype (Supplementary Fig. S7). There was a significant correlation in the log linear model (*r*^2^ = 0.578, *p* = 0.0174). Two patients with the Cys/Cys genotype experienced mild or severe leukopenia at the dose of 6 MP of 5 mg/day, but the times to leukopenia were 42 days (mild leukopenia without alopecia) and 101 days (severe leukopenia with severe alopecia). The former patient was able to continue being treated with 6 MP for 1029 days by adjusting the dose from 2 to 5 mg/day. From these results, there is a possibility that the patients with the Cys/Cys genotype can start 6 MP at 1–2 mg/day. However, considering the purpose of thiopurines in IBD, there is a question about why the patients need to try thiopurines and thus a risk of severe AEs. By taking into account these results, we can make provisional recommendations regarding the safe initial dose of thiopurines. These doses are AZA at 50 mg/day or 6 MP at 30 mg/day in Japanese adult IBD patients with the genotype of Arg/Arg or Arg/His, AZA at 25 mg/day or 6 MP at 10–15 mg/day in patients with Arg/Cys, 6 MP at 5–10 mg/day in patients with Cys/His, and thiopurines are contraindicated in patients with the Cys/Cys genotype (Supplementary Table 10).

There are several limitations in this study. First, this was a retrospective multicenter study, so the methods of thiopurine usage and monitoring of AEs varied. In particular, time to leukopenia depended on how frequently WBC counts were examined. Second, the sample size of AEs except for leukopenia and alopecia was very small, and the results of GWASs for other AEs such as pancreatitis were very limited. Finally, the analysis was performed only in the Japanese patients with IBD. Therefore, the selection of predictive models and recommendation of the optimal doses were applicable only to Japanese patients with IBD.

In conclusion, a multicenter pharmacogenetic study revealed that the genotyping of NUDT15 codon 139 is the best way to predict severe leukopenia and alopecia in Japanese patients with IBD. We provided provisional recommendations on safe initial doses of thiopurines according to the genotype of the patient. However, further prospective study is required to evaluate these recommendations.

## Electronic supplementary material

Below is the link to the electronic supplementary material.

Supplementary material 1 (PDF 2188 kb)

## Abbreviations

IBD: Inflammatory bowel disease
UC: Ulcerative colitis
CD: Crohn’s disease
BD: Behçet’s disease
AZA: Azathiopurine
6-MP: 6-Mercaptopurine
AE: Adverse event
GWAS: Genome-wide association study
HWE: Hardy–Weinberg equilibrium
PCA: Principal component analysis
QC: Quality control
SNP: Single-nucleotide polymorphism
ROC: Receiver operating characteristic
WBC: White blood cell
