# Supplementary material for: NUDT15 codon 139 is the best pharmacogenetic marker for predicting thiopurine-induced severe adverse events in Japanese patients with inflammatory bowel disease: a multicenter study
Source: J Gastroenterol. 2018 Jun 19;53(9):1065–78. doi: 10.1007/s00535-018-1486-7 (PMC6132901; doi:10.1007/s00535-018-1486-7)
Supplement: Supplementary file 1 — Supplementary material 1 (PDF 2188 kb) [file 535_2018_1486_MOESM1_ESM.pdf]

Supplementary Tables & Figures

Supplementary Figure Legends

Supplementary Figure S1. Haplotype structures of NUDT15 and frequencies in the Japanese

Haplotypes *1–*6 of NUDT15 were defined from the combination of four non-synonymous variants [1]. The haplotypes containing three additional variants from a recent study [2] and two novel variants found in this study [3] were defined as *7– *11. All of these novel haplotypes were very rare in the Japanese population.

Supplementary Figure S2 Manhattan plots for results of the discovery and conditional GWASs for thiopurine-induced severe leukopenia and severe alopecia

Single-nucleotide polymorphisms are plotted according to chromosomal location, with the $-\log_{10}(P)$ from the results of GWASs. The red line indicates the threshold for the genome-wide significance ($P = 1E^{-8}$). The blue line indicates the threshold for nominal significance ($P = 1E^{-6}$). (A) GWAS for thiopurine-induced severe leukopenia (WBC < 2000/μL), (B) conditional GWAS for severe leukopenia on rs116855232 (p.Arg139Cys), (C) GWAS for acute severe leukopenia (WBC < 2000/μL, <8 weeks), (D) conditional GWAS for acute severe leukopenia on rs116855232, (E) GWAS for severe alopecia, and (F) conditional GWAS for severe alopecia on rs116855232. All significant associations disappeared in the conditional GWASs.

Supplementary Figure S3. Locus zoom plots of p-values around the top-hit NUDT15 region from the GWAS result with thiopurine-induced leukopenia. (A) The top associated SNP, rs116855232, is shown as purple diamonds and the remaining SNPs are shown as circles, with color indicating the level of linkage disequilibrium (R2) with rs116855232. (B) Plots of p-values from the conditional analysis on rs116855232. All associations disappeared.

Supplementary Figure S4. Manhattan plots for results of the GWASs for thiopurine-induced AEs.

Single-nucleotide polymorphisms are plotted according to chromosomal location, with the −log10(P) from the results of GWASs. The red line indicates the threshold for the genome-wide significance ($P = 1E^{-8}$). The blue line indicates the threshold for nominal significance (P = 1E−6). (A) GWAS for thiopurine-induced pancreatitis, (B) GWAS for infection, (C) GWAS for digestive symptoms, (D) GWAS for liver dysfunction, (E) GWAS for skin symptoms, and (F) GWAS for fever.

Supplementary Figure S5. (A)(B) Locus zoom plots of p-values around two candidate loci from the GWAS result with thiopurine-induced pancreatitis. The top associated SNPs in each locus are shown as purple diamonds and the remaining SNPs are shown as circles, with color indicating the level of linkage disequilibrium (R2) with lead SNP.

Supplementary Figure S6. ROC curve of predictive models for thiopurine-induced AEs

ROC analyses were performed to compare the predictive logistic regression models in combination with NUDT15 codon 139 or diplotype, ABCC4 and RUNX1.

AUCs of each model to predict the AEs were evaluated. There was a significant difference between the model NUDT15_Codon139 and NUDT15_Haplotype in leukopenia (WBC < 3000/μL); there was no significant difference between the models in other severe AEs.

Supplementary Figure S7. Correlation between 6-MP doses and time to leukopenia in the patients with the codon 139 genotype of Cys/Cys. The log linear model was used to evaluate the correlation; significant associations were observed.

References

1. Moriyama T, Nishii R, Perez-Andreu V, Yang W, Klussmann FA, Zhao X, et al. NUDT15 polymorphisms alter thiopurine metabolism and hematopoietic toxicity. Nat Genet. 2016;48(4):367-73.
2. Moriyama T, Yang YL, Nishii R, Ariffin H, Liu C, Lin TN, et al. Novel variants in NUDT15 and thiopurine intolerance in children with acute lymphoblastic leukemia from diverse ancestry. Blood. 2017;130(10):1209-12.
3. Nagasaki M, Yasuda J, Katsuoka F, Nariai N, Kojima K, Kawai Y, et al. Rare variant discovery by deep whole-genome sequencing of 1,070 Japanese individuals. Nat Commun. 2015;6:8018.

Supplementary Table 1. Amplification primers for direct sequencing of NUDT15 coding regions

Exons of NUDT15	Primers	
Exon 1	Forward	TGTAAAACGACGGCCAGTATATTCAAAGCACAACTGTAAGCGACT
	Reverse	CAGGAAACAGCTATGACCCGTTCTCACGCACGGCACA
Exon 2	Forward	TGTAAAACGACGGCCAGTGAATTAATCTAATTTTTGTTTCTGTTTTCCA
	Reverse	CAGGAAACAGCTATGACCAGCATTCTCTTCATATGGCAACAT
Exon 3	Forward	TGTAAAACGACGGCCAGTGTTAGCTTACCCAAATAAACACCCTTTG
	Reverse	CAGGAAACAGCTATGACCTTCATTCCCTAACCAGACCTTATTCTTG

Supplementary Table 2. Patient characteristics and genotype frequencies of NUDT15 codon 139

	SASP	5-ASA	IFX	ADA	Thiopurines	Total (IBD)	2KJPN
**Number of subjects**	322	2461	1028	491	1291	**2627**	**2036**
**Disease**							
UC	218 (67.7%)	1448 (58.8%)	337 (32.8%)	170 (34.6%)	722 (55.9%)	**1519 (57.8%)**	**-**
CD	101 (31.4%)	970 (39.4%)	666 (64.8%)	307 (62.5%)	548 (42.4%)	**1048 (39.9%)**	**-**
BD	3 (0.9%)	43 (1.7%)	25 (2.4%)	14 (2.9%)	21 (1.6%)	**60 (2.3%)**	**-**
**Age at study entry**							
Mean (range)	48.3 (4-89)	40.5 (3-89)	38.6 (3-80)	38.6 (10-83)	41.0 (3-84)	**40.6 (3-89)**	**-**
**Sex**							
Male	190 (59.0%)	1528 (62.1%)	673 (65.5%)	309 (62.9%)	819 (63.4%)	**1647 (62.7%)**	**-**
Female	132 (41.0%)	933 (37.9%)	355 (34.5%)	182 (37.1%)	472 (36.6%)	**980 (37.3%)**	**-**
**Adverse events**							
	40 (12.4%)	197 (8.0%)	124 (12.1%)	27 (5.5%)	454 (35.2%)	**-**	**-**
**Codon 139**							
Arg/Arg	242 (75.2%)	1900 (77.2%)	791 (76.9%)	372 (75.8%)	958 (74.2%)	**2026 (77.1%)**	**1651 (81.1%)**
Arg/Cys	65 (20.2%)	496 (20.2%)	201 (19.6%)	102 (20.8%)	275 (21.3%)	**534 (20.3%)**	**362 (17.8%)**
Cys/Cys	15 (4.7%)	56 (2.3%)	32 (3.1%)	16 (3.3%)	49 (3.8%)	**56 (2.1%)**	**22 (1.1%)**
Arg/His	0 (0.0%)	6 (0.24%)	4 (0.39%)	1 (0.20%)	7 (0.54%)	**8 (0.30%)**	**1 (0.05%)**
Cys/His	0 (0.0%)	3 (0.12%)	0 (0.0%)	0 (0.0%)	2 (0.15%)	**3 (0.11%)**	**0 (0.0%)**

Supplementary Table 3. Case–control association studies between the genotypes of p.Arg139Cys and AEs associated with IBD drugs

IBD drugs	Genotype frequencies* (% to each AE group)						p-values**	Allelic association*** (Arg vs. Cys)	
	AE(+)			AE(−)					
	Arg/Arg	Arg/Cys	Cys/Cys	Arg/Arg	Arg/Cys	Cys/Cys		p-values	OR (95%CI)
**SASP**	27 (67.5%)	11 (27.5%)	2 (5.0%)	215 (76.2%)	54 (19.1%)	13 (4.6%)	0.325	0.363	1.40 (0.76–2.57)
**5-ASA**	163 (82.7%)	31 (15.7%)	3 (1.5%)	1737 (76.7%)	465 (20.5%)	53 (2.3%)	0.068	0.070	0.71 (0.50–1.01)
**IFX**	95 (76.6%)	26 (21.0%)	3 (2.4%)	696 (77.0%)	175 (19.4%)	29 (3.2%)	0.986	1.000	1.00 (0.67–1.48)
**ADA**	23 (85.2%)	4 (14.8%)	0 (0.0%)	349 (75.2%)	98 (21.1%)	16 (3.4%)	0.192	0.240	0.49 (0.17–1.38)
**thiopurines**	260 (57.3%)	141 (31.1%)	49 (10.8%)	698 (83.4%)	134 (16.0%)	0 (0.0%)	$1.29E^{-32}$	$1.55E^{-36}$	4.13 (3.28–5.20)

AE: adverse event, OR: odds ratio, CI: confidence interval

* Rare genotypes (CH and RH) were excluded

** Cochran–Armitage trend analysis

*** Chi-squared test

Supplementary Table 4. Patient characteristics from thiopurine study and breakdown of adverse events

Disease	UC	CD	BD	p-values*	Total
**Number of subjects**	722	548	21	-	**1291**
**Gender (M/F)**	425 / 297	382 / 166	12 / 9	-	**819 / 472**
**Adverse Events of Thiopurines**	256 (35.5%)	193 (35.2%)	5 (23.8%)	0.60	**454 (35.2%)**
**Leukopenia (WBC < 3000/μL)**	129 (17.9%)	104 (19.0%)	3 (14.3%)	0.83	**236 (18.3%)**
**Alopecia**	54 (7.5%)	32 (5.8%)	1 (4.8%)	0.48	**87 (6.7%)**
**Liver Dysfunction**	33 (4.6%)	13 (2.4%)	1 (4.8%)	0.09	**47 (3.6%)**
**Pancreatitis**	13 (1.8%)	7 (1.3%)	0 (0.0%)	0.64	**20 (1.5%)**
**Digestive symptoms**	56 (7.8%)	37 (6.8%)	0 (0.0%)	0.46	**93 (7.2%)**
**Infection**	10 (1.4%)	7 (1.3%)	0 (0.0%)	1.00	**17 (1.3%)**
**Fever**	6 (0.8%)	7 (1.3%)	0 (0.0%)	0.66	**13 (1.0%)**
**Skin symptoms**	3 (0.4%)	4 (0.7%)	0 (0.0%)	0.53	**7 (0.5%)**
**Malignant tumor**	1 (0.1%)	1 (0.2%)	0 (0.0%)	1.00	**2 (0.2%)**

UC: ulcerative colitis, CD: Crohn's disease, BD: intestinal Behçet's disease

*Fisher's exact tests

Supplementary Table 5. Top hit regions from the results of GWASs for thiopurine-induced AEs

**Adverse Events**	**CHR**	**From* (Mbp)**	**To* (Mbp)**	**SNPs ** **	**Genes*****	**Top SNP**				
						dbSNP	Ref	Alt	P-value	OR (95%CI)_
Leukopenia	Chr3	62.87	62.87	1	CADPS	rs117829974	C	T	1.62E-07	2.1 (1.6–2.7)
(WBC < 3000/μL)	**Chr13**	**48.24**	**48.73**	**365**	**NUDT15, SUCLA2, MED4**	**rs116855232**	**C**	**T**	**1.32E-33**	**5.8 (4.4–7.7)**
Alopecia	Chr5	125.68	125.68	1	GRAMD3	rs75466870	A	T	5.07E-07	4.5 (2.5–8.0)
	Chr6	47.08	47.08	1	GPR110, TNFRSF21, GPR116	rs117927258	T	C	1.86E-07	6.5 (3.2–13.2)
	Chr10	96.42	96.42	1	HELLS, CYP2C18, CYP2C19, TBC1D12	rs145080284	C	G	9.73E-07	3.8 (2.2–6.5)
	Chr11	42.15	42.15	1		rs78844412	T	A	5.97E-07	6.9 (3.2–14.8)
	**Chr13**	**48.42**	**48.73**	**336**	**NUDT15, SUCLA2, MED4**	**rs116855232**	**C**	**T**	**4.26E-29**	**10.4 (6.9–15.6)**
Fever		no candidate			-	-	-	-	-	-
Liver Dysfunction		no candidate			-	-	-	-	-	-
Digestive Symptoms	Chr1	90.31	90.31	1	LRRC8D, LRRC8C, ZNF326	rs12035735	G	A	6.59E-07	5.0 (2.7–9.6)
Infection		no candidate								
Liver Dysfunction		no candidate								
Pancreatitis	Chr3	2.06	2.08	6	CNTN4	rs4437130	G	A	1.77E-07	6.8 (3.3–14.1)
	Chr9	98.32	98.32	3	PTCH1	rs62561366	A	T	5.24E-07	7.0 (3.3–14.8)
Skin Symptoms		no candidate			-	-	-	-	-	-

*Positions are based on the Genome Reference Consortium human build 37 (GRCh37)

**Number of SNPs with P-values $< 1 \times 10^{-6}$

***Genes located within the region ± 200 kbp

Supplementary Table 6. Conditional GWASs of leukopenia and severe alopecia on NUDT15 Arg139Cys

**Adverse Events**	**CHR**	**From* (Mbp)**	**To* (Mbp)**	**SNPs ****	**Genes***	**Top SNP**				
						dbSNP	Ref	Alt	P-value	OR (95%CI)_
Leukopenia	Chr1	212.84	212.84	1	FAM71A,BATF3,ATF3,(3)	rs2501846	T	C	9.91E-07	1.94 (1.49–2.52)
Severe Leukopenia		no candidate			-	-	-			
Acute Severe Leukopenia	Chr2	196.01	196.14	6	-	rs117506642	C	A	4.76E-07	13.3 (4.9–36.3)
Alopecia		no candidate			-	-	-	-	-	-
Severe Alopecia		no candidate			-	-	-	-	-	-

*Positions are based on the Genome Reference Consortium human build 37 (GRCh37)

**Number of SNPs with P-values < 1×10^{-6}

***Genes located within the region ± 200 kbp

Supplementary Table 7. Association of thiopurine-induced leukopenia (WBC < 3000/μL) with previously reported variants

Chr	Position*	dbSNP	Gene / Location	A1	A2	A1 Frequencies		p-values**	OR (95%CI)	p-values** on rs116855232
						Control (n=1024)	Case (n=196)			
6	18130918	rs1142345	TPMT A719G (Tyr240Cys)	G	A	2.5%	1.0%	2.12E-01	0.40 (0.09–1.69)	4.22E-01
13	48619855	rs116855232	NUDT15 C415T (Arg139Cys)	T	C	17.9%	75.0%	**1.32E-33**	5.80 (4.36–7.71)	NA
13	95815415	rs3765534	ABCC4 G2269A (Glu757Lys)	A	G	31.3%	22.4%	**2.30E-02**	0.67 (0.48–0.95)	7.09E-02
16	53860052	rs79206939	FTO G400A (Ala134Thr)	A	G	4.4%	3.6%	6.20E-01	0.82 (0.37–1.82)	7.48E-01
20	3193842	rs1127354	ITPase C94A(Pro32Thr)	A	C	27.6%	27.6%	9.92E-01	1.00 (0.73–1.38)	7.58E-01
21	36564651	rs2834826	RUNX1 upstream	T	C	72.3%	82.7%	5.35E-02	1.24 (1.00–1.55)	**1.66E-02**

*Positions are based on the Genome Reference Consortium human build 37 (GRCh37)

**Logistic regression model with sex as a covariate

Supplementary Table 8. Evaluation of different prediction models of leukopenia and severe alopecia using NUDT15 variants.

**Model**	**Cut-off**		**Leukopenia (WBC < 3000/μL)**			**Severe Leukopenia (WBC < 2000/μL)**			**Acute Severe Leukopenia (WBC < 2000/μL. < 8 weeks)**			**Severe Alopecia**		
	Negative	Positive	Sensitivity	Specificity	AUC	Sensitivity	Specificity	AUC	Sensitivity	Specificity	AUC	Sensitivity	Specificity	AUC
**Codon139_A**	RR	RC,CC,RH,CH	0.566	0.809	0.687	0.806	0.782	0.794	0.923	0.772	0.848	1.000	0.771	0.885
**Codon139_B**	RR,RH	RC,CC,CH	0.560	0.816	0.688	0.806	0.790	**0.798***	0.923	0.780	**0.852***	1.000	0.778	0.889
**Codon139_C**	RR,RH,RC	CC,CH	0.200	0.997	0.599	0.448	0.992	0.720	0.667	0.991	0.829	0.919	0.997	0.958
**Codon139_D**	RR,RH,RC,CH	CC	0.189	0.997	0.593	0.418	0.992	0.705	0.641	0.992	0.817	0.919	0.999	**0.959***
**Diplotype_A**	NN	NI,NL,IL,LL	0.594	0.787	**0.691***	0.821	0.759	0.790	0.923	0.749	0.836	1.000	0.747	0.874
**Diplotype_B**	NN,NI	NL,IL,LL	0.566	0.816	**0.691***	0.806	0.788	0.797	0.923	0.779	0.851	1.000	0.777	0.889
**Diplotype_C**	NN,NI,NL	IL,LL	0.206	0.995	0.600	0.448	0.989	0.718	0.667	0.988	0.827	0.919	0.994	0.956
**Diplotype_D**	NN,NI,NL,IL	LL	(Same as Codon139_D Model)											

AUC: Area under Receiver Operating Characteristic (ROC) curve, NN: Normal and Normal (1*1), NI: Normal and Intermediate (*1*4,*1*5,*1*6), NL: Normal and Low (*1*2,*1*3*,1*9), IL: Intermediate and Low (*2*4,*2*5,*3*4,*3*5), LL: Low and Low (*2*2,*2*3,*3*3)

*Best AUC in each adverse event.

Supplementary Table 9. Number of subjects in GWASs

Adverse Events	Case	Control
Leukopenia	196	1024
Severe Leukopenia	72	1148
Acute Severe Leukopenia	43	1172
Alopecia (all)	81	1139
Severe Alopecia	41	1179
Pancreatitis	18	1202
Fever	10	1210
Digestive Symptoms	93	1127
Infection	14	1206
Liver dysfunction	39	1181
Skin symptoms	6	1214

Supplementary Table 10. Provisional recommendations of safe initial doses of thiopurines in terms of the genotype of NUDT15 codon 139

Genotype of Codon 139	Risk of severe adverse events if the patients keep taking normal dose of thiopurines		Estimated Safe Initial Dose
	Acute Severe Leukopenia	Severe alopecia	
**Arg/Arg**	Rare (<0.1%)	Rare (<0.1%)	AZA 50 mg/day or 6-MP 30 mg/day
**Arg/His**	Rare (<0.1%)	Rare (<0.1%)	AZA 50 mg/day or 6-MP 30 mg/day
**Arg/Cys**	Low risk (<5%)	Low risk (<5%)	AZA 25 mg/day or 6-MP 10–15 mg/day
**Cys/His**	High risk (>50%)	Rare (<0.1%)	6-MP 5–10 mg/day
**Cys/Cys**	Inevitable	Inevitable	Contraindication (6-MP 1–2 mg/day?)

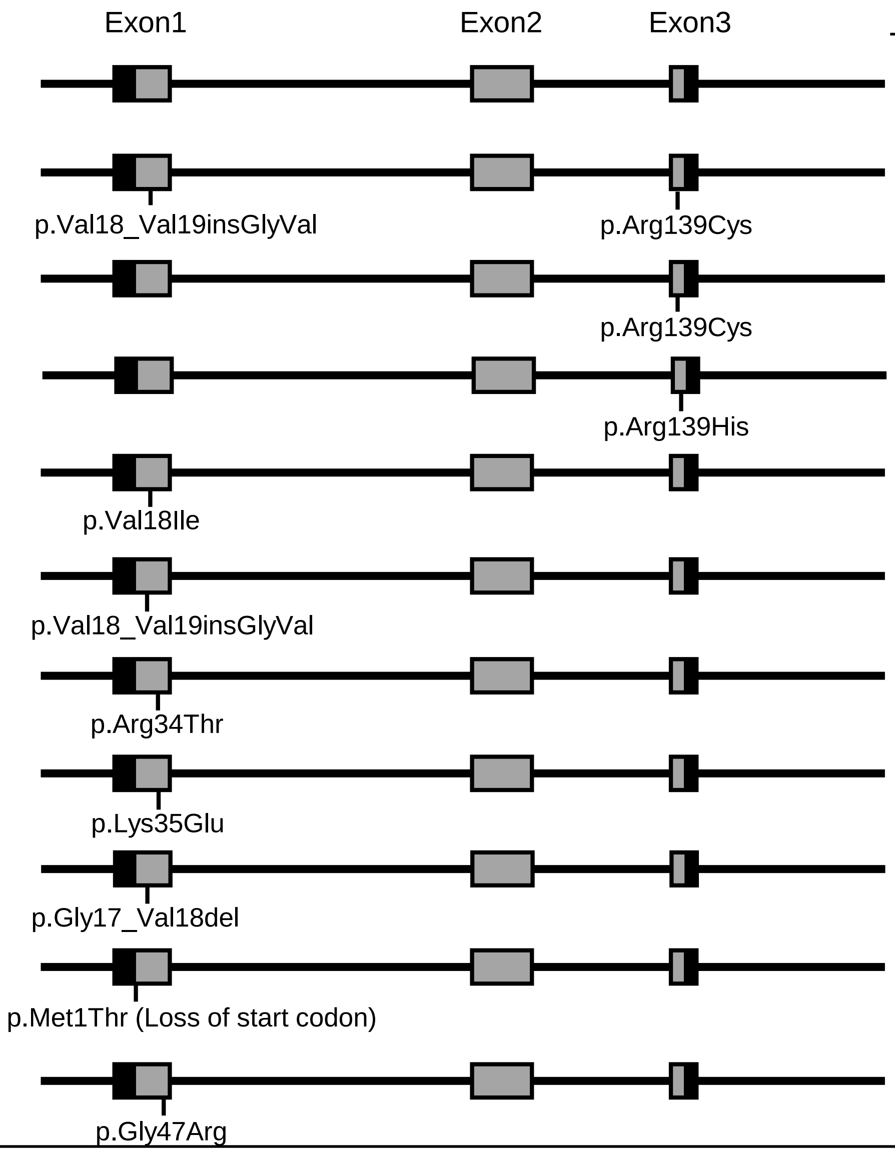

NUDT15 haplotypes	Variant	Enzyme activity in vitro	Reference	Haplotype frequencies 2KJPN (n = 2036)	MENDEL (n = 970)
*1		Normal	Moriyama et al [1]	3625 (89.0%)	1627 (83.9%)
*2	p.Val18_Val19insGlyVal; p.Arg139Cys	Low	Moriyama et al [1]	126 (3.1%)	101 (5.2%)
*3	p.Arg139Cys	Low	Moriyama et al [1]	280 (6.9%)	178 (9.2%)
*4	p.Arg139His	Intermediate	Moriyama et al [1]	1 (0.025%)	9 (0.46%)
*5	p.Val18Ile	Intermediate	Moriyama et al [1]	33 (0.81%)	20 (1.0%)
*6	p.Val18_Val19insGlyVal	Intermediate	Moriyama et al [1]	4 (0.10%)	4 (0.21%)
*7	p.Arg34Thr	Low	Moriyama et al [2]	0 (0.0%)	0 (0.0%)
*8	p.Lys35Glu	Intermediate	Moriyama et al [2]	0 (0.0%)	0 (0.0%)
*9	p.Gly17_Val18del	Low	Moriyama et al [2]	1 (0.025%)	1 (0.052%)
*10	p.Met1Thr (Loss of start codon)	-	Nagasaki et al [3]	1 (0.025%)	0 (0.0%)
*11	p.Gly47Arg	-	Nagasaki et al [3]	1 (0.025%)	0 (0.0%)

Supplementary Figure S1

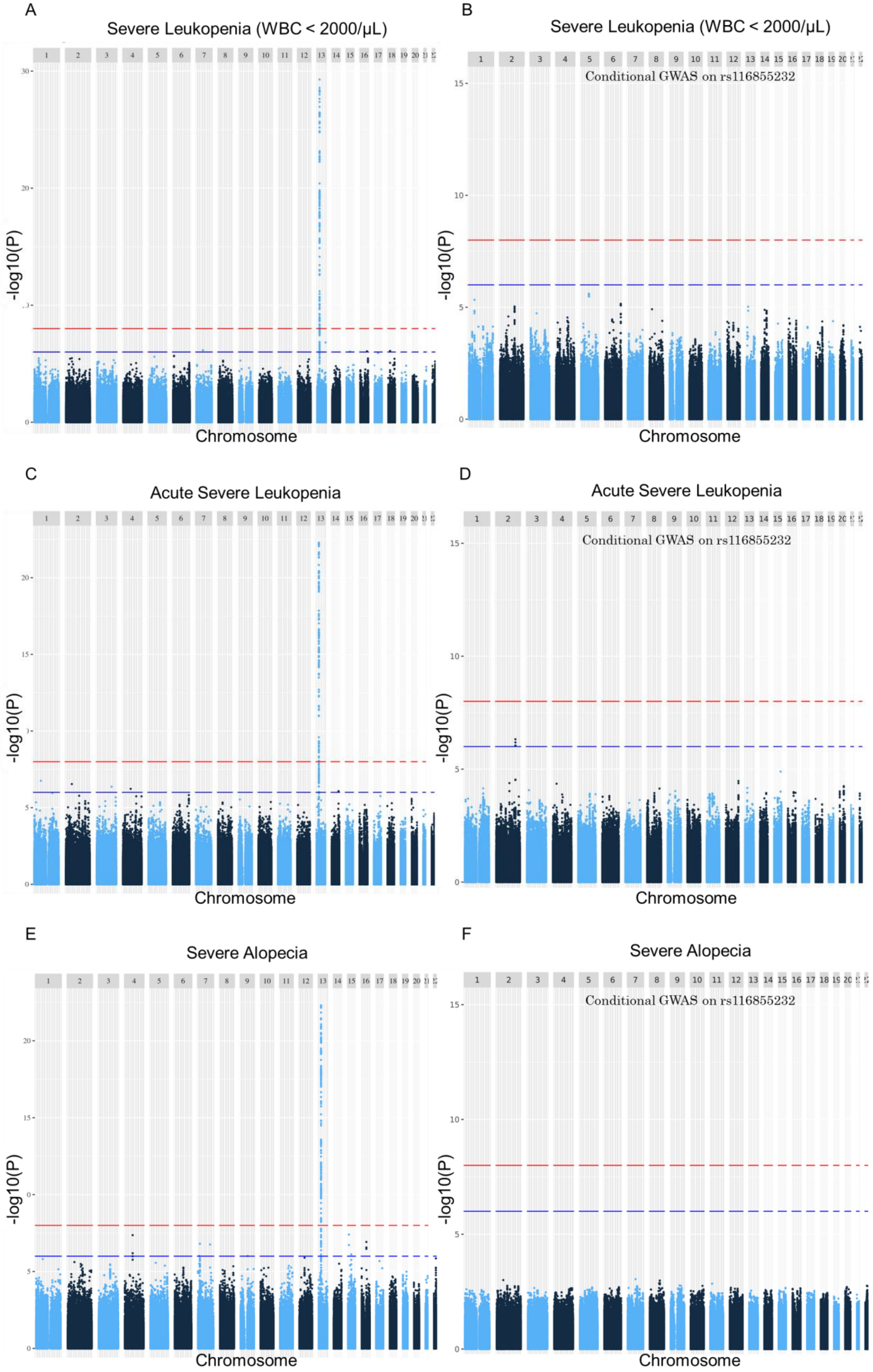

Supplementary Figure S2

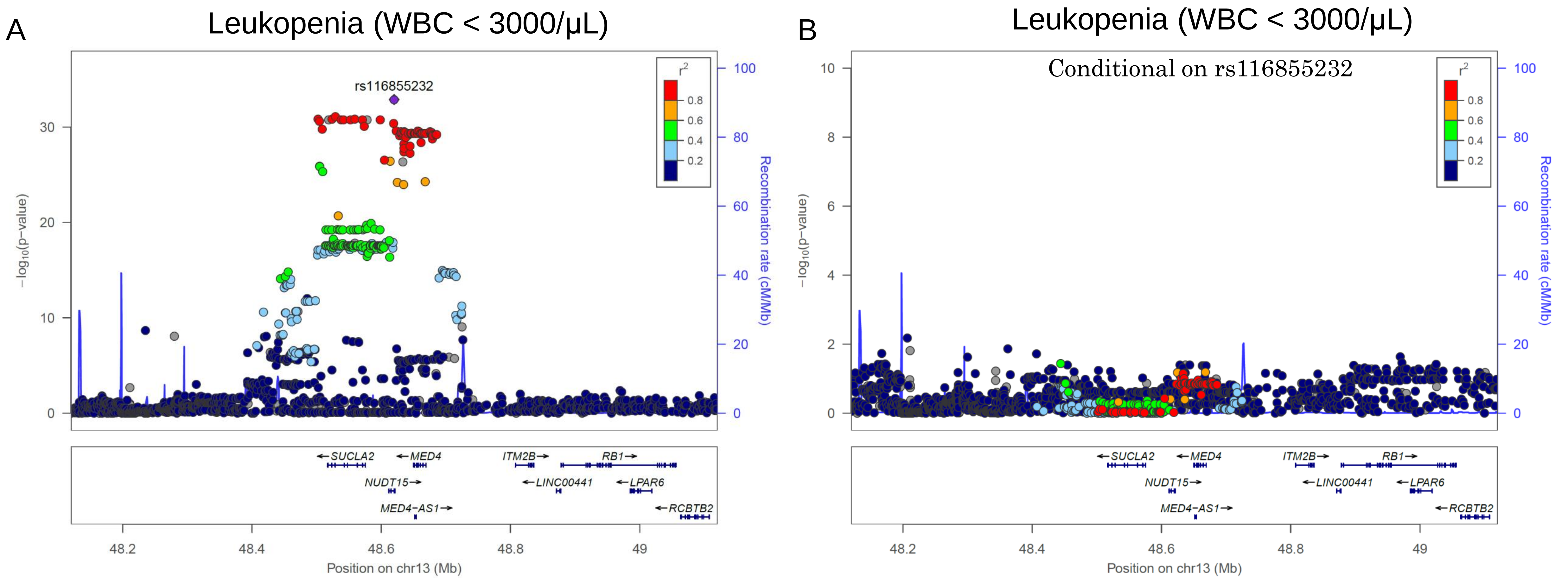

Supplementary Figure S3

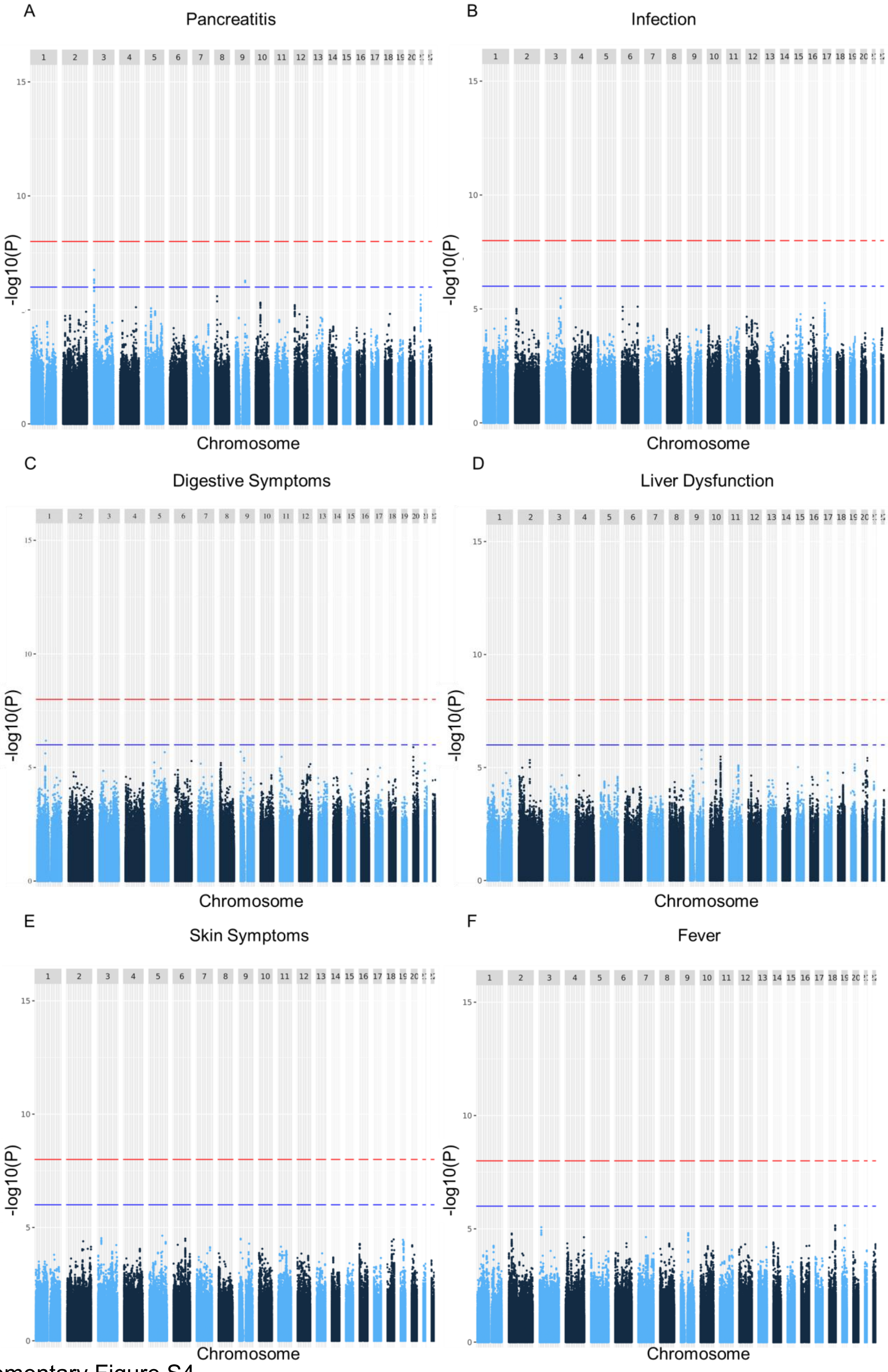

Supplementary Figure S4

A

Pancreatitis

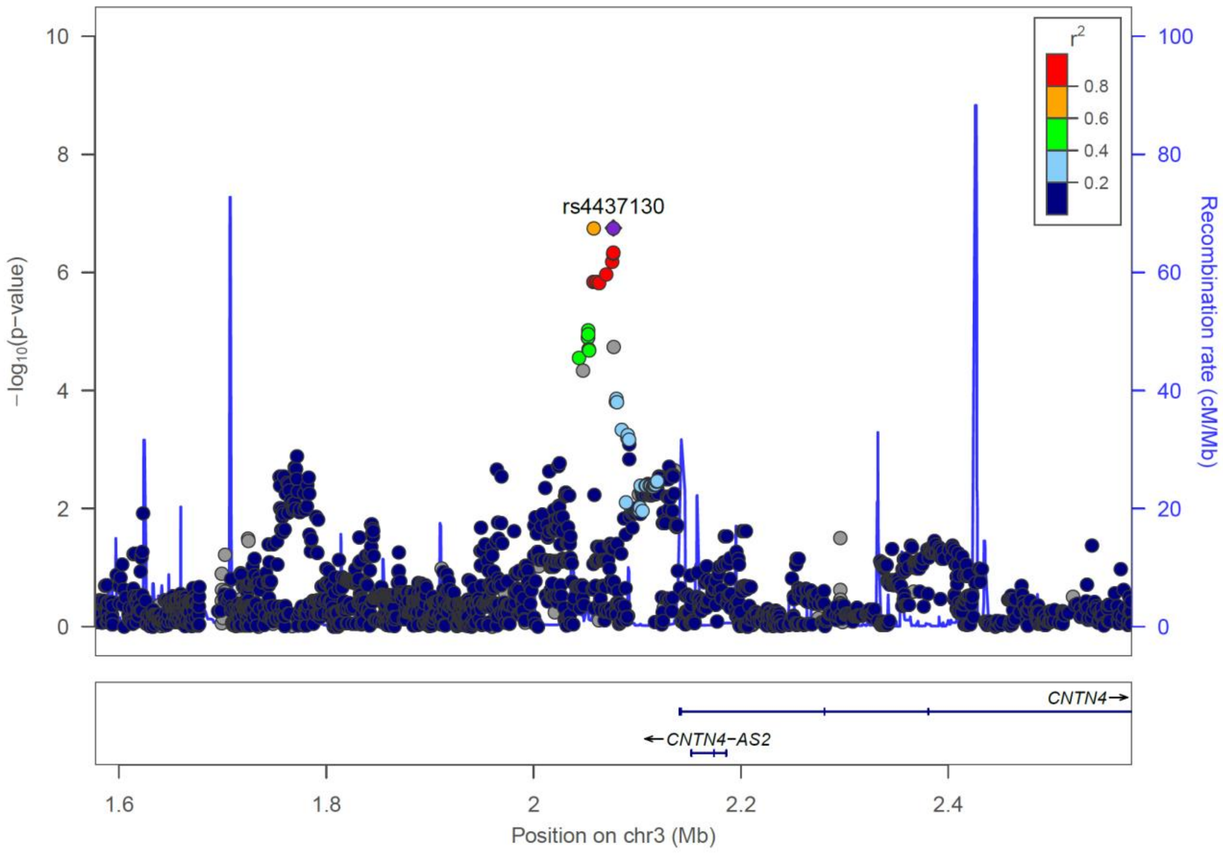

B

Pancreatitis

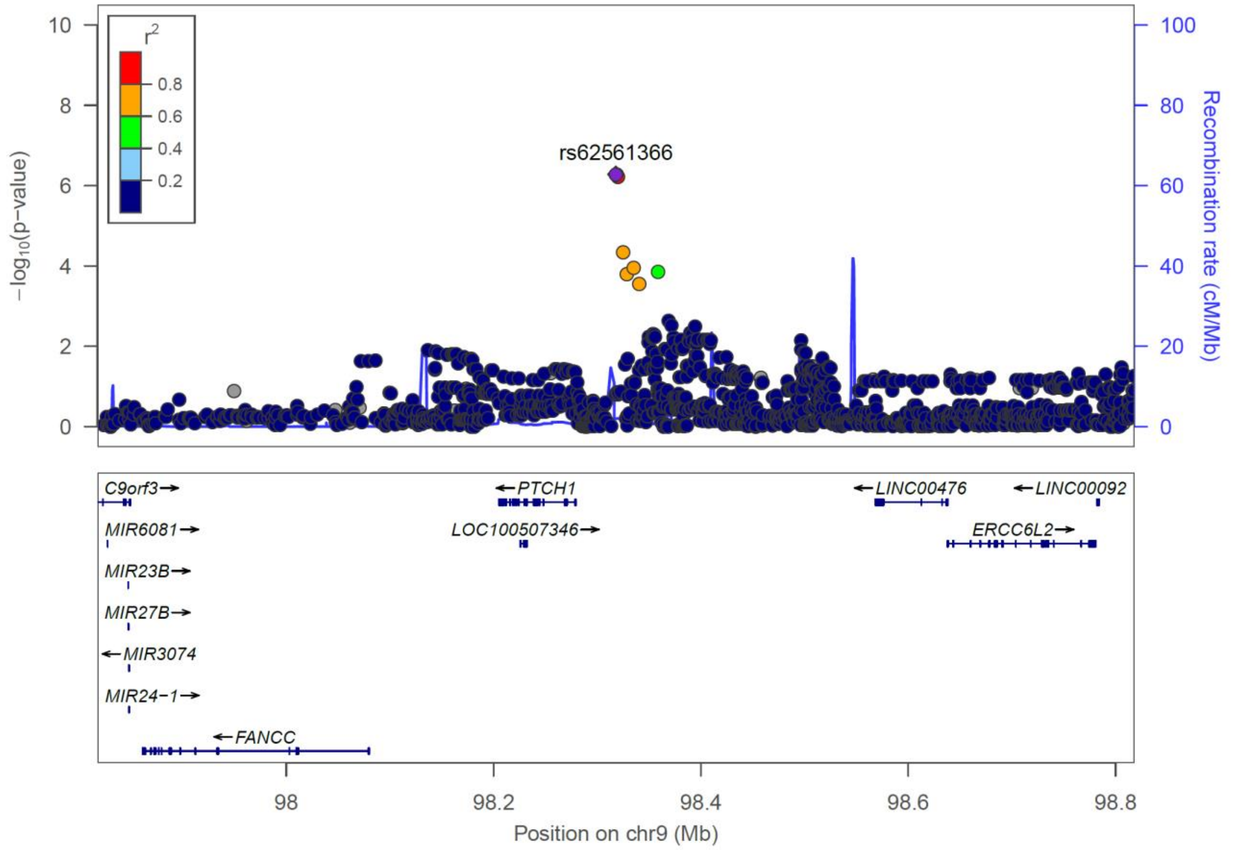

Supplementary Figure S5

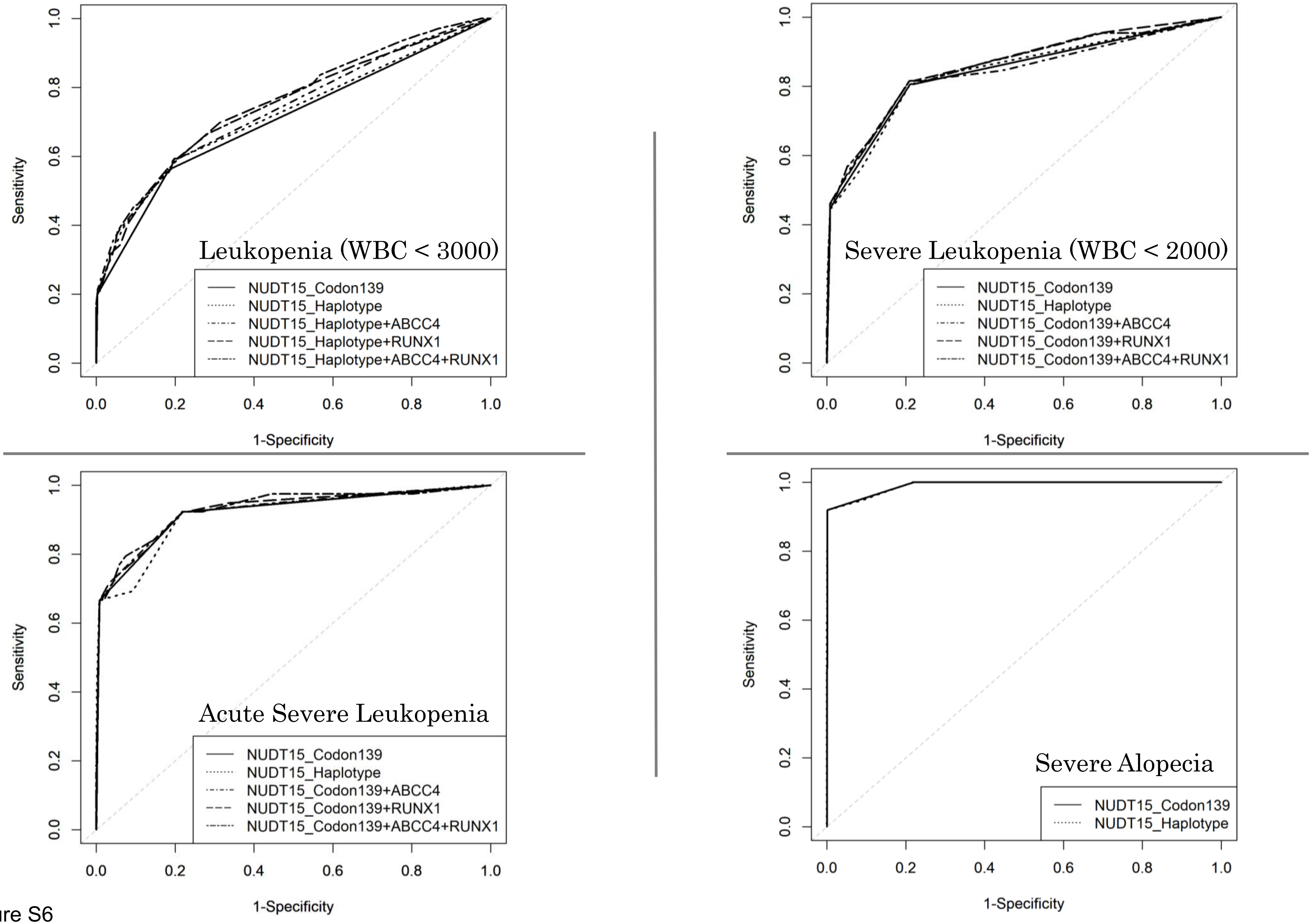

Supplementary Figure S6

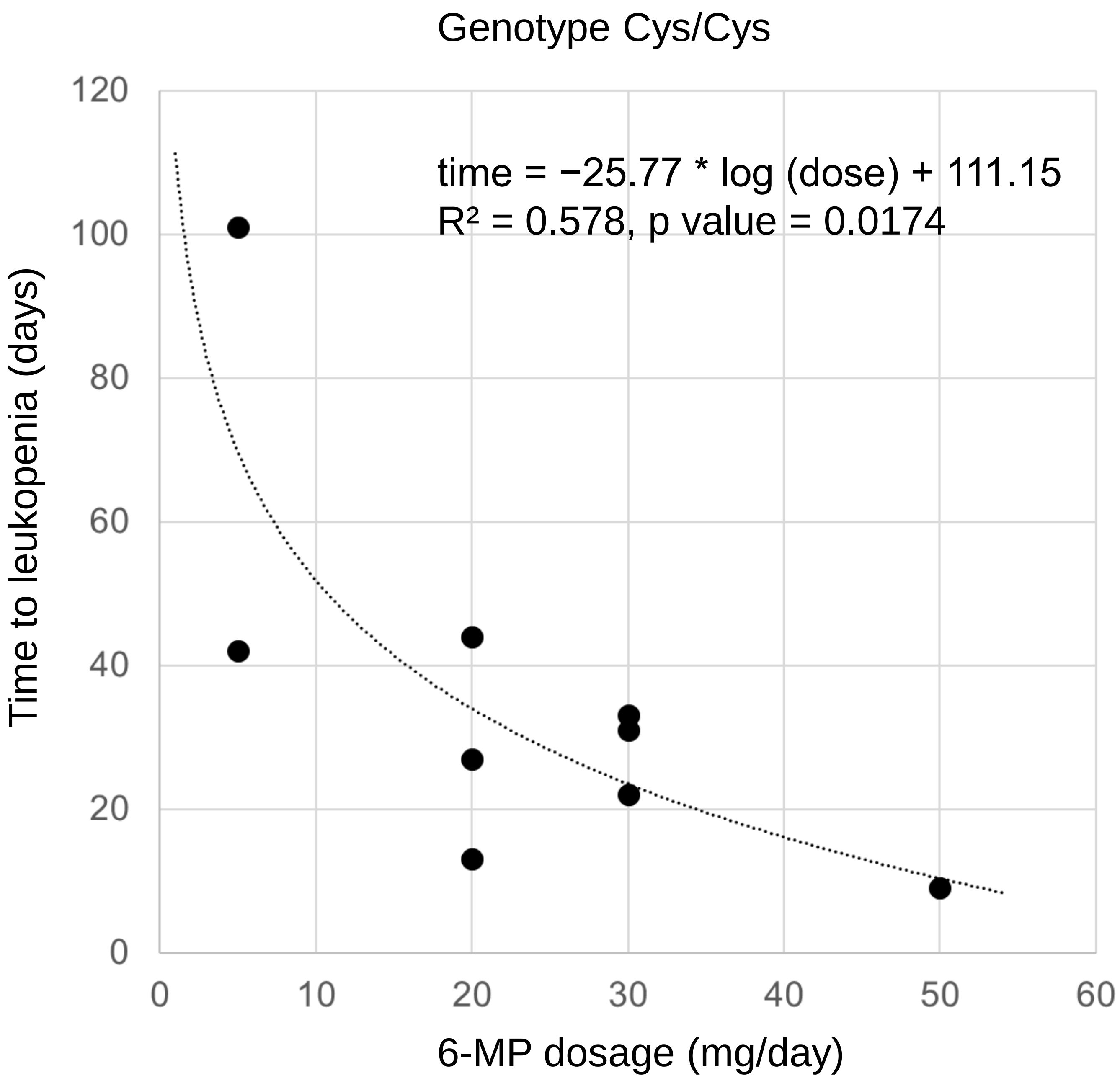

Supplementary Figure S7
